# N-dependent dynamics of root growth and nitrate and ammonium uptake are altered by the bacterium *Herbaspirillum seropedicae* in the cereal model *Brachypodium distachyon*

**DOI:** 10.1093/jxb/erac184

**Published:** 2022-05-05

**Authors:** Weiqi Kuang, Stefan Sanow, Jana M Kelm, Mark Müller Linow, Peter Andeer, Dietrich Kohlheyer, Trent Northen, John P Vogel, Michelle Watt, Borjana Arsova

**Affiliations:** College of Life and Environmental Sciences, Hunan University of Arts and Science, 415000 Changde, China; Key Laboratory of Agro-Ecological Processes in Subtropical Region, Institute of Subtropical Agriculture, Innovation Academy for Seed Design, Chinese Academy of Sciences, 410125 Changsha, China; IBG-2 Plant Sciences, Institut für Bio- und Geowissenschaften, Forschungszentrum Jülich, Wilhelm-Johnen-Straße, 52428 Jülich, Germany; IBG-2 Plant Sciences, Institut für Bio- und Geowissenschaften, Forschungszentrum Jülich, Wilhelm-Johnen-Straße, 52428 Jülich, Germany; IBG-2 Plant Sciences, Institut für Bio- und Geowissenschaften, Forschungszentrum Jülich, Wilhelm-Johnen-Straße, 52428 Jülich, Germany; Environmental Genomics and Systems Biology, Lawrence Berkeley National Laboratory, Berkeley, CA, USA; IBG-1 Biotechnology, Institut für Bio- und Geowissenschaften, Forschungszentrum Jülich, Wilhelm-Johnen-Straße, 52428 Jülich, Germany; Environmental Genomics and Systems Biology, Lawrence Berkeley National Laboratory, Berkeley, CA, USA; The Joint Genome Institute, Lawrence Berkeley National Laboratory, Berkeley, CA, USA; Environmental Genomics and Systems Biology, Lawrence Berkeley National Laboratory, Berkeley, CA, USA; The Joint Genome Institute, Lawrence Berkeley National Laboratory, Berkeley, CA, USA; IBG-2 Plant Sciences, Institut für Bio- und Geowissenschaften, Forschungszentrum Jülich, Wilhelm-Johnen-Straße, 52428 Jülich, Germany; Faculty of Science, The University of Melbourne, Melbourne, Australia; IBG-2 Plant Sciences, Institut für Bio- und Geowissenschaften, Forschungszentrum Jülich, Wilhelm-Johnen-Straße, 52428 Jülich, Germany; National Research Council, Italy

**Keywords:** Agriculture, *Brachypodium*, cereals, crop productivity, EcoFAB, *Herbaspirillum seropedicae*, N fixation, nitrogen, non-invasive phenotyping, plant-growth promoting rhizobacteria, wheat

## Abstract

Nitrogen (N) fixation in cereals by root-associated bacteria is a promising solution for reducing use of chemical N fertilizers in agriculture. However, plant and bacterial responses are unpredictable across environments. We hypothesized that cereal responses to N-fixing bacteria are dynamic, depending on N supply and time. To quantify the dynamics, a gnotobiotic, fabricated ecosystem (EcoFAB) was adapted to analyse N mass balance, to image shoot and root growth, and to measure gene expression of *Brachypodium distachyon* inoculated with the N-fixing bacterium *Herbaspirillum seropedicae*. Phenotyping throughput of EcoFAB-N was 25–30 plants h^−1^ with open software and imaging systems. *Herbaspirillum seropedicae* inoculation of *B. distachyon* shifted root and shoot growth, nitrate versus ammonium uptake, and gene expression with time; directions and magnitude depended on N availability. Primary roots were longer and root hairs shorter regardless of N, with stronger changes at low N. At higher N, *H. seropedicae* provided 11% of the total plant N that came from sources other than the seed or the nutrient solution. The time-resolved phenotypic and molecular data point to distinct modes of action: at 5 mM NH_4_NO_3_ the benefit appears through N fixation, while at 0.5 mM NH_4_NO_3_ the mechanism appears to be plant physiological, with *H. seropedicae* promoting uptake of N from the root medium.Future work could fine-tune plant and root-associated microorganisms to growth and nutrient dynamics.

## Teaser

The nitrogen-fixing bacterium *Herbaspirillum seropedicae*altered growth, nitrate or ammonium uptake, and gene expression of *Brachypodium distachyon*dynamically in specialized, fabricated ecosystems. Nitrogen fertilization and time were the most important variables.

## Introduction

Nitrogen (N) fertilizer use has been increasing since the industrial revolution, reaching 145.5 million metric tons worldwide in 2018 (www.fertilizerseurope.com). In some countries laws have placed stringent limits on the application of N fertilizers, with the aims of minimizing the leaching that leads to eutrophication of freshwater resources, decreasing environmental impact of fertilizer production (Bundesministerium der Justiz und für Verbraucherschutz, 2017), and reducing emissions into the atmosphere and climate warming. To minimize synthetic fertilizer use but sustain and improve plant performance to meet population demand, the following are required (i) increased N_2_ fixation from the atmosphere and N uptake into the root through N-fixing bacteria (Glick, 2010, 2012), and (ii) optimized use of N within the plant.

The cereals wheat, maize, and rice represent 70% of global crops. In cereals, the global use efficiency of nitrogen fertilizer increased only 2% in the 13 years between 2002 and 2015, from 33% to 35%. The gain is attributed to agronomy using precision agriculture and breeding of more N-efficient varieties (Omara *et al.*, 2019). Nitrogen is taken up by cereal roots through a system of transporters on the root cell plasma membrane with high or low affinity for N-containing ions (ammonium and nitrate), and the activation of each system is dependent on the environmental conditions (O’Brien *et al.*, 2016). Notably, plant roots exist in a dynamic environment, with temporal changes in growth and anatomy, expression of nutrient transporters that is root type and age dependent, and constant interaction with soil and microorganisms of varying quality (Arsova *et al.*, 2020).

Root-associated bacteria that live within or on the roots in the rhizosphere are increasingly being developed for commercial use in cereal agriculture (de Souza *et al.*, 2015). However, the exact mode of action is often not understood (Dent and Cocking, 2017) and performance in field crops is unpredictable and inconsistent (Pii *et al*., 2015), with growth effects ranging from negative to positive across global wheat trials. To fully exploit the potential of N-fixing root-associated bacteria, we need to understand the developmental and molecular modes of action of members of the grass family, *Poaceae*, over time.

We use the model grass *Brachypodium dystachion*, with similar but smaller genome (Brkljacic *et al.*, 2011), similar root development (Watt *et al.*, 2009), and similar microbiome (Kawasaki *et al.*, 2016) to cereals, along with *Herbaspirillum seropedicae*, an endophytic diazotroph that has been shown to colonize *B. distachyon* (do Amaral *et al.*, 2016). *Herbaspirillum* spp. are found in roots, stems, and leaves of many plants, most without disease symptoms (Roncato-Maccari *et al.*, 2003), and can promote the growth of *Poaceae* (maize, *Michantus*, rice, sorghum, and sugarcane; Monteiro *et al.*, 2012). Specifically, *H. seropedicae* increased growth and nitrogen accumulation in rice varieties (Divan-Baldani *et al.*, 2000; Gyaneshwar *et al.*, 2002; Roncato-Maccari *et al.*, 2003; Trovero *et al.*, 2018). In light of the variable plant growth-promoting rhizobacterial response found in the field, we hypothesized that *B. distachyon*’s response to *H. seropedicae* will be dynamic, will vary depending on the environment the plants are grown in (in our case with varying N availability), and in addition, will change through time.

A recently established fabricated ecosystem, EcoFAB (Gao *et al.*, 2018), joins a palette of microfluidic devices developed to study plant response to various conditions (Massalha *et al.*, 2017). Importantly, EcoFAB was shown to be a reproducible system in a ring-trial that followed not only phenotypic but also metabolomic traits across four laboratories on two continents (Sasse *et al.*, 2019). EcoFABs remain gnotobiotic over time, with reproducible *B. distachyon* root and shoot phenotypes under different P supply (Sasse *et al.*, 2019). To quantify and resolve plant–microorganism dynamics in the *B. distachyon*–*H. seropedicae* phenotype, we modified the EcoFAB to supply different N levels to the plant (from here on EcoFAB-N), and quantified N uptake and imaged roots and shoots non-invasively over time. Non-invasive phenotyping allows the acquisition of temporal and spatial plant parameters from the time microorganisms are introduced to a system until a pre-determined point in plant growth (Schillaci *et al*, 2021). Non-invasive plant phenotyping has been rapidly extended to include mobile, inexpensive, and portable methods, such as use of mobile phones (Müller-Linow *et al.*, 2019), promoting applicability in labs around the world. So, our second hypothesis states that a gnotobiotic system in combination with non-invasive imaging has the potential to achieve temporal resolution of the plant response to microbes, on the tissue, molecular, and nutritional levels. Thus, we combined non-invasive measures with uptake of different N forms by plants from the root medium, with repeated biomass and N sampling and transcript analysis at two destructive time points, to resolve the dynamics of the plant–bacterial phenotype for N fixation in cereals.

## Materials and methods

### EcoFAB-N devices

EcoFAB-N devices were fabricated as published (Gao *et al.*, 2018) with modifications to achieve N-deficient and -sufficient conditions. Pilot testing indicated that the original EcoFABs were not large enough for low N conditions (Supplementary Fig. S1). Additionally, the 3D printed mold (used for polymerization of the EcoFAB chamber) was simplified into one unit for easier reproduction. The resulting volume of the EcoFAB-N chamber was 4 ml. See ‘Design and preparation of EcoFAB-N’ (doi: dx.doi.org/10.17504/protocols.io.b53tq8nn) for full details of the EcoFAB-N production. Each EcoFAB-N was housed in a rectangular plastic box (95 × 65 × 65 mm) (Modulor, cat. no. 0216662, Germany), with holes (diameter 5 mm) drilled on both sides for aeration. The holes were covered with micropore tape to maintain sterility (3M, cat. no. 1530-0). The EcoFAB-N units were washed, sterilized, and filled with medium as described in Sasse *et al.*, (2019).

### Plant growth conditions

*Brachypodium distachyon* Bd21-3 (Vogel and Hill, 2008) was prepared and introduced into the EcoFAB-N as described (Sasse *et al.*, 2019). ­Seedlings were germinated on 0.8% phyto agar medium, pH 6 (Duchefa Biochemie, cat. no. P1003.1000). After 2 d germination, seedlings with comparable size were picked to conduct the experiment, transplanted to an EcoFAB-N, and incubated in a 16 h light–8 h dark regime at 24 °C, with 150 µE illumination. To each of the 44 EcoFAB-N chambers, 4 ml of high nitrogen (5 mM NH_4_NO_3_) or low nitrogen (0.5 mM NH_4_NO_3_) medium was added (composition: 0.625 mM KH_2_PO_4_, 3 mM MES, 0.05 mM FeSO_4_·H_2_O, 0.05 mM Na_2_EDTA·2H_2_O, 1.5 mM CaCl_2_, 0.75 mM MgSO_4_·7H_2_O, 0.025 mM H_3_BO_3_, 0.005 Mm MnSO_4_·H_2_O, 0.001 mM ZnSO_4_·7H_2_O, 0.0025 mM KI, 0.0001 mM Na_2_MoO_4_·2H_2_O, 0.00005 mM CoCl_2_·6H_2_O, 0.0005 mM CuSO_4_·5H_2_O, pH adjusted to 5.8). Media were changed and collected at 4, 8, 11, 14, 17, 20, 23, and 26 d after transfer (DAT) and the plants were harvested at 11, 20, and 28 DAT.

### Bacterial propagation and inoculation

**Fig. 1.**
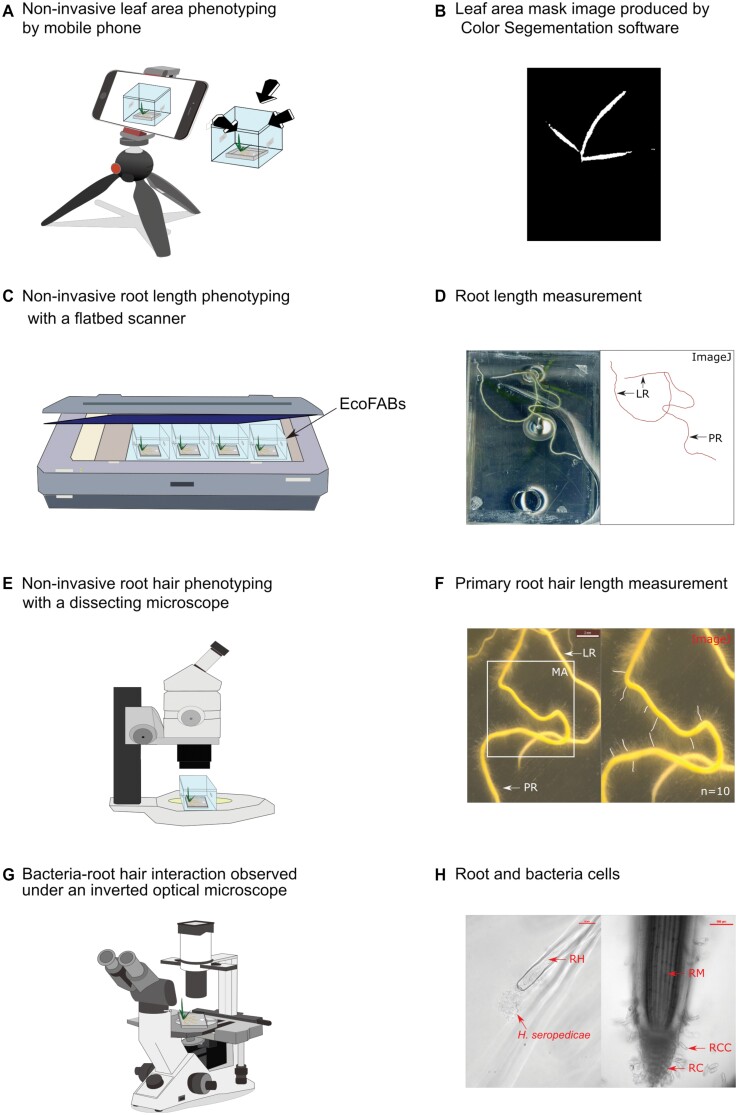
Non-invasive approaches to phenotyping shoots and roots of *B. distachyon* growing in EcoFABs. (A) Shoot images (leaves) were taken with a mobile phone using Plant Screen Mobile software (Müller-Linow *et al.*, 2019) over time. The EcoFAB-N was positioned at three angles around the mobile phone on the clean bench. (B) Images from (A) were transferred to computer and binarized from a masked image generated by Color Segmentation software (Müller-Linow *et al.*, 2015); the resulting project leaf area data in pixels were calibrated and converted to units of cm^2^ using Eqs 1, 2 (see ‘Material and methods’). (C) Root length and type were obtained from images of multiple EcoFABs positioned directly on a glass, back-lit flatbed scanner. (D) Root system images were analysed with the ImageJ software for primary roots (PR) and lateral roots (LR) total length manually. (E) Root hairs were observed and images were taken with a dissecting microscope. (F) Hairs on primary root were measured between the root outer edge and the tips of hairs from the measurement area (MA): 1–4 mm (4 DAT), 2–8 mm (11 DAT), 2–10 mm (17 DAT), and 4–15 mm (23 and 28 DAT), using ImageJ software manually, *n*=10 hairs per root. (G) Cellular level imaging was done on EcoFABs mounted on an optical inverted microscope. (H) *Herbaspirillum seropedicae* associated with a *B. distachyon* root hair (RH, left), and root cap cells (RCC) around a root cap (RC, right) were observed through the bases of the EcoFABs.

*Herbaspirillum seropedicae* Z67 (DSM 6445) was purchased from the German Collection of Microorganisms and Cell Cultures GmbH (DSMZ) and confirmed by sequencing the 16S rRNA gene using 27F and 1492R primers (Supplementary Table S1). The bacteria were recovered and propagated in 30 ml lysogeny broth (LB) medium (Sigma, cat. no. L3022) for 2 d. The bacteria were centrifuged and resuspended in the same volume of EcoFAB-N medium twice and the OD was adjusted to 0.6 before inoculation (40 µl) into the EcoFAB-N chamber (22 EcoFABs at high nitrogen and 22 EcoFABs at low nitrogen) at 4 DAT. Bacteria had 4 d to colonize the roots. With the next medium exchange at 8 DAT, beside nutrient-depleted medium, we removed bacteria that were not physically attached to the plant root. Sterility of the EcoFAB-N with control medium (22 EcoFABs at high nitrogen and 22 EcoFABs at low nitrogen) was tested by plating out aliquots onto LB medium (Sasse *et al.*, 2019). Contaminated EcoFAB-N units were excluded from analysis. Live imaging of bacterial colonization of the plant root (Fig 1G, H) can be performed as described under doi: dx.doi.org/10.17504/protocols.io.b53uq8nw.

### Shoot phenotyping

Non-invasive leaf area phenotyping was developed for the EcoFAB-N to allow shoot growth dynamic variation through time (Fig. 1A, B; see ‘Non-invasive imaging of leaf area using a mobile phone in the EcoFAB-N’ doi: dx.doi.org/10.17504/protocols.io.b53kq8kw, for detailed method). Briefly a mobile phone (Huawei p20) at a resolution of 1920 × 1080 pixels (px) adjusted by Plant Screen Mobile (Müller-Linow *et al.*, 2019) was used to capture leaf area within the EcoFAB-N box at 4, 8, 11, 14, 17, 20, 23, 26, and 28 DAT. Images obtained in pixels were calibrated with destructive leaf area and biomass measurements (Supplementary Fig. S2). Images were processed and analysed with Color Segmentation software by HSV threshold with setting, hue: 48-134; saturation: 28-119; intensity: 60-192 (Müller-Linow *et al.*, 2015). The leaf area in pixels was converted to cm^2^ using:


$$\begin{array}{l} Plant\;leaf\;area\;\left(cm^{2}\right)\;=\;{\left[\frac{Picture\;\;\;width\;\;\;(cm)}{Picture\;\;\;width\;\;\;(px)}\right]}^{2\;}\times \;plant\;leaf\;area\;\left(px\right) \end{array}$$ (1)

Data were calibrated to eliminate picture difference due to phone angle or distance interference using:


$$\begin{array}{l} Calibrated\;leaf\;area\;=\;plant\;leaf\;area\;\left(cm^{2}\right)\;/\;{\left[\frac{Width\;\;\;of\;\;\;EcoFAB\;\;\;(px)}{Width\;\;\;of\;\;\;EcoFAB\;\;\;mean\;\;\;(px)}\right]}^{2} \end{array}$$ (2)

The minimum number of photo shots required was evaluated between three, five and eight photo shots using a mobile camera positioned at a 45° angle facing downwards. The camera positioning is depicted in Supplementary Fig. S2A, B.

**Fig. 2.**
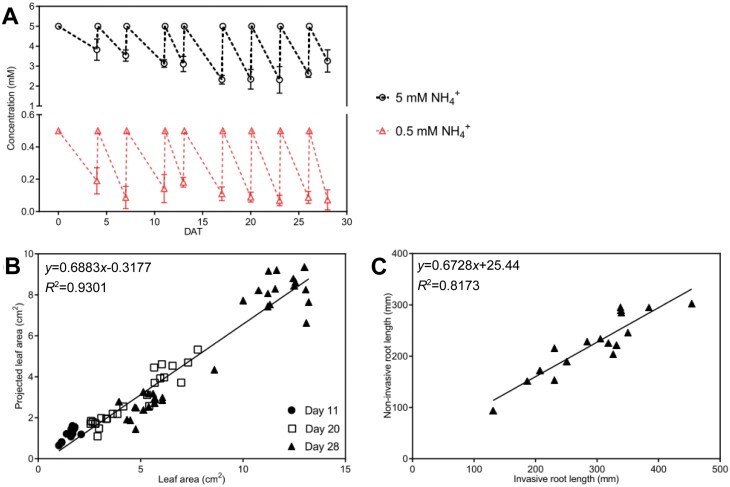
Verification of *B*. *distachyon* non-invasive shoot and root phenotyping and nitrogen supply in EcoFAB-N. (A) Ammonium depletion and replenishment in EcoFAB-N medium. *Brachypodium distachyon* were grown in revised 0.5 MS medium with 5 mM NH_4_NO_3_ (5 mM NH_4_^+^) or 0.5 mM NH_4_NO_3_ (0.5 mM NH_4_^+^). Medium was changed regularly starting from 4 DAT, until harvest at 28 DAT. Ammonium concentration was measured by continuous flow analysis (*n*=5). (B) Correlation between non-invasive leaf area measured (Fig. 1A, B) and invasive leaf area measured by leaf area meter Li 3100 at 11, 20, and 28 DAT (*n*=12, 24, and 32 respectively). (C) Correlation between non-invasive total root length imaged by flatbed scanner and analysed by ImageJ (Fig. 1C, D), and invasive total root length imaged by flatbed scanner and measured by WinRHIZO software, at 28 DAT (*n*=17). Data (A) are means ±standard error. DAT, days after transplanting to EcoFAB-N.

For invasive phenotyping, plant shoots were harvested on DAT 11, 20, and 28. Fresh and dry weight were determined for individual shoots. Additionally, leaf area was invasively determined using a leaf area meter (Li3100, LI-COR). The correlation between invasive and non-invasive leaf area acquisition was analysed using information from 11, 20, and 28 DAT (Fig. 2B).

### Root phenotyping

Root system and root hair measurements were obtained at 4, 11, 17, 20, 23, and 28 DAT. Roots were scanned (Epson 10000XL) and root hairs were measured using a stereomicroscope (Leica, MZ12) inside the EcoFAB-N chambers (Fig. 1C–F). Depending on the developmental stage, root hairs were measured in a pre-decided region from the tip of the primary root, specifically 1–4 mm (4 DAT), 2–8 mm (11 DAT), 2–10 mm (17 DAT), and 4–15 mm (23 and 28 DAT). Later the images were analysed by ImageJ (version 2.0.0) and total root length, primary root length and root hair length were analysed through time. Lateral root length was calculated by subtracting the primary root length from the total root length.

Invasive root phenotyping was performed at 11, 20, and 28 DAT. Root fresh and dry weight were determined on a subsection of the available plants, listed in the respective figure legends. At 28 DAT, five roots from each group were scanned (Epson 10000XL) and binarized with WinRHIZO (Regent Instruments Inc., Canada), for determination of root length and comparison to non-invasive imaging.

### Nitrogen measurement and mass balance calculation

The seeds, shoot, and roots from harvested plants (11 DAT (*n*=3), 20 DAT (*n*=3), and 28 DAT (*n*=5)), were dried and ground into powder. Then total nitrogen was analysed with a CHNS analyser (Vario EL Cube, Elementar) with thermal conductivity detection. The medium in EcoFAB-N was collected and the nitrate concentration was determined by ion chromatography (Dionex ICS-3000, Thermo Scientific) using column AS14 with eluent 1 mM NaHCO_3_, 8 mM Na_2_CO_3_, and column AS23 with eluent 0.8 mM NaHCO_3_, 4.5 mM Na_2_CO_3_. The ammonium concentration was determined by continuous flow analysis (CFA Analyser FLOWSYS 3-Kanal, Alliance Instruments GmbH). The nitrogen (ammonium and nitrate) depletion in EcoFAB-N over time was calculated as follows:


$$\begin{array}{l} N{H_{4}}^{+}\;depletion=\left(C_{ai}-C_{af}\right)\times V \end{array}$$ (3)

$$\begin{array}{l} N{O_{3}}^{-}\;depletion=\left(C_{ni}-C_{nf}\right)\times V \end{array}$$ (4)

$$\begin{array}{l} N{H_{4}}^{+}\;cumulative\;depletion=\underset{DAT\;1}{\overset{DAT\;28}{\sum }}\;\;\left[\left(C_{ai}-C_{af}\right)\times V\right] \end{array}$$ (5)

$$\begin{array}{l} N{O_{3}}^{-}\;cumulative\;depletion=\underset{DAT\;1}{\overset{DAT\;28}{\sum }}\;\;\left[\left(C_{ni}-C_{nf}\right)\times V\right] \end{array}$$ (6)

where *C*_ni_ is the initial nitrate concentration in the medium, *C*_nf_ is the final nitrate concentration in the medium, *C*_ai_ is the initial ammonium concentration in the medium, *C*_af_ is the final ammonium concentration in the medium, and *V* is the medium volume in EcoFAB. Finally, the cumulative nitrogen depletion (CND) was compared with the total nitrogen in the plant:


$$\begin{array}{ll} CND=\; & C_{s}\times W_{s}+N{H_{4}}^{+}\;cumulative\;depletion\\ & +N{O_{3}}^{-}\;cumulative\;depletion \end{array}$$ (7)

where *C*_s_ is the average nitrogen concentration in the seed and *W*_s_ is the average dry weight of the seed.

### Quantitative real-time PCR

Roots and shoots from three plants, were harvested from each group at 20 and 28 DAT. Tissue was separately frozen in liquid nitrogen and stored at −80 °C. Frozen tissue was ground in a ball-mill (Retsch MM200, Retsch, Germany) with a steel ball (Retsch, cat. no. 22.455.0001). Total RNA was extracted from 45 mg shoot tissue powder or 35 mg root tissue powder using the RNeasy Plant Mini Kit (Qiagen, Germany) according to the manufacturer’s protocol. To remove genomic DNA contamination, total RNA was incubated with DNAse I (Thermo Fisher Scientific, AM1906). The cDNA was synthesized using 350 ng of total RNA. First-strand cDNA was synthesized using SuperScript IV reverse transcriptase (Thermo Fisher Scientific, cat. no. 18090050). The absence of genomic DNA contamination was verified with the housekeeping gene *SamDc* (Supplementary Table S1). For quantitative real-time PCR, 100 ng first-strand cDNA (50 ng μl^−1^) was mixed with 0.6 µl of 10 µM solution of the primer pair of interest, together with 10 µl iQ SYBR Green Supermix (Bio-Rad, cat. no. 1708884), and water to a total volume of 20 µl. The PCR was performed using an qPCR Bio-Rad CFX Connect system employing the following program: 95 °C for 2 min, followed by 35 cycles of 95 °C for 10 s, 55 °C for 10 s, and 72 °C for 20 s. The primers used for the genes of interest are listed in Supplementary Table S1.

For data analyses, one specific template and primer pair (in six technical replicates) was used as an interplate calibration on each plate. The interplate calibration was used to normalize the *C*_q_ values between the different runs as described in Ståhlberg *et al.* (2013). Then, the Δ*C*_q_ and ΔΔ*C*_q_ values were calculated as described in Rao *et al.* (2013).

The housekeeping gene used was *UBQ10* (Supplementary Table S1) and results were compared with the expression in 5 mM NH_4_NO_3_ for each specific tissue and time point. The variation of *UBQ10* was within 1 *C*_q_ for each tissue and time point. It is worth noting that while we tried to use a second housekeeping gene (and tested *SamDC*), the combination of nutrient availability, growth stages, and presence/absence of bacterium made the expression too variable, rendering it unsuitable for this purpose.

## Results

### EcoFAB-N adaptations for nitrogen uptake quantification and shoot and root non-invasive imaging

Enlargement of the EcoFAB-N to 4 ml from 2.8 ml (Sasse *et al.*, 2019) allowed maintenance of N within physiological levels (Fig. 2A) (David *et al.*, 2019), and relieved the sharp depletion at high (2.5 mM NH_4_NO_3_) and low N (0.25 mM NH_4_NO_3_) (Supplementary Fig. S1) in the original 2.8 ml chamber, which led to large variability in the nutrient conditions, particularly as plants became larger (20 DAT). We increased the volume of the chamber to 4 ml by maximizing the chamber space on top of the microscope slide (doi: dx.doi.org/10.17504/protocols.io.b53tq8nn; Fig. 1A), and set the N supply to 0.5 and 5 mM NH_4_NO_3_ (Fig. 2A). In the process the mold frame and base were designed as a single body to exclude leakage of polydimethylsiloxane (doi: dx.doi.org/10.17504/protocols.io.b53tq8nn). This shortened the cleaning process and reduced the fabrication time by half an hour (Gao *et al.*, 2018).

Non-invasive shoot phenotyping was performed from various angles to account for the architectural variability of the *B. distachyon* shoots and ensure that the EcoFAB-N box-enclosure did not interfere with the imaging process (Fig. 1A, B). After comparing a number of imaging scenarios (with three, five, and eight images per plant (Supplementary Fig. S2A, B), we established that three images were sufficient for reliable projected leaf area (PLA) determination. The *R*^2^ value in the correlation between 20 and 28 DAT projected leaf area versus invasive leaf area was 0.924 in the three-image scenario and only 0.03 higher at the eight-image scenario (Supplementary Fig. S2B). The PLA estimation had the necessary dynamic range for the growing plants using the three-image approach and comparing the PLA with invasive leaf area (Fig. 2B) and fresh weight at 11, 20, and 28 DAT (Supplementary Fig. S2C). We found reasonable linear correlations for leaf area (*R*^2^=0.9301) (Fig. 2B) or shoot fresh weight (*R*^2^=0.9279) (Supplementary Fig. S2C) with PLA. Thus, the biological interpretation of leaf area was performed without additional calibration of the PLA.

Root image acquisition using the scanner with a blue background placed on top of the EcoFAB-N boxes (Fig. 1C, D) overcame shoot interference that arose from imaging from the top (as in Sasse *et al.*, 2019). Roots were visible despite varying coverage from the growing shoot, and up to 12 EcoFAB-N were imaged at once. Comparison of the non-invasive total root length (Fig. 1C, D) and invasive total root length measured by WinRHIZO at 28 DAT resulted in a linear correlation with *R*^2^=0.817 (Fig. 2C). Thus, the biological interpretation of projected root length was performed without additional calibration of the projected root length.

### Growth of shoots and roots of *B. distachyon* after inoculation with *H. seropedicae*

**Fig. 3.**
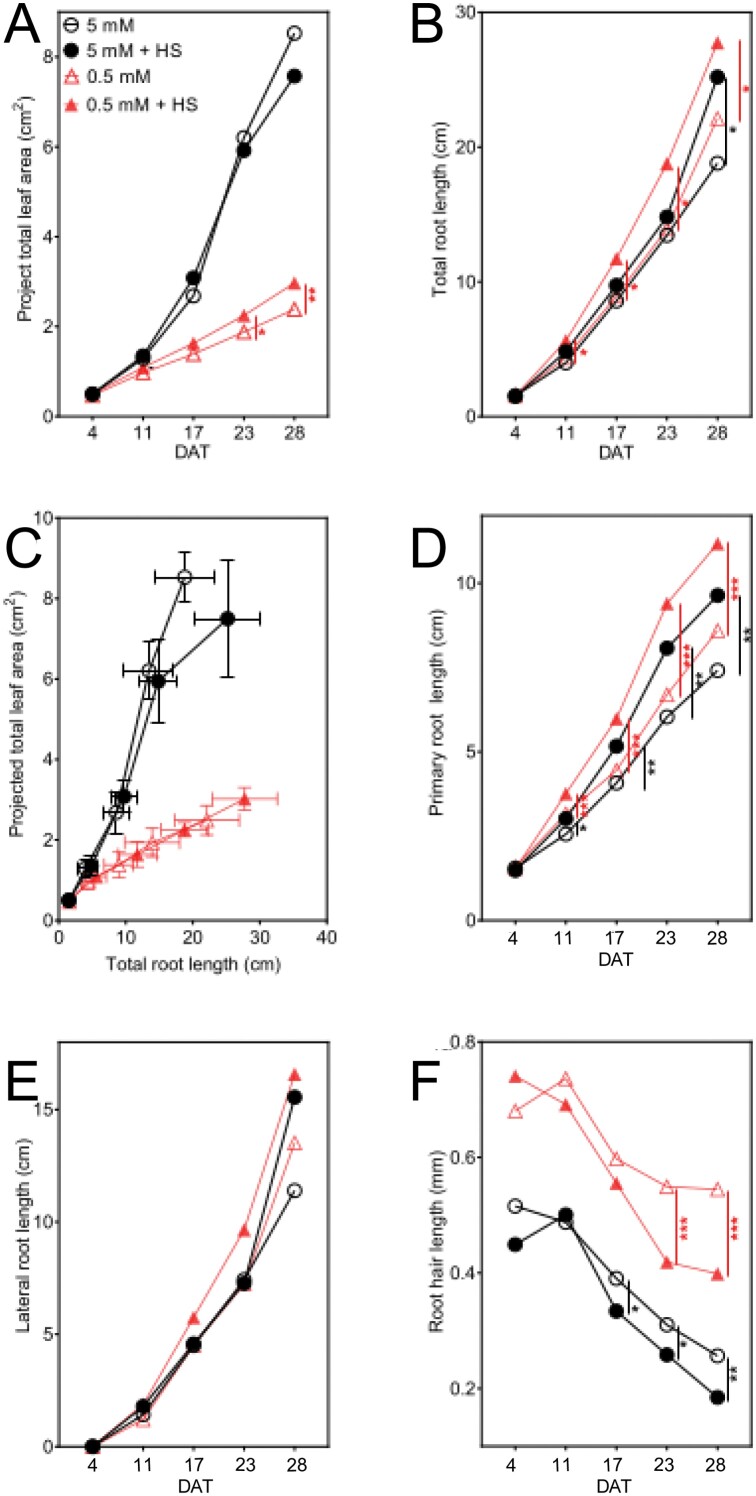
Growth of *B. distachyon* measured non-destructively over time at 4, 11, 17, 23, and 28 DAT when roots exposed to 5 mM NH_4_NO_3_ or 0.5 mM NH_4_NO_3_, with or without *H*. *seropedicae* (HS), in EcoFAB-N chambers in Fig. 1. (A) Projected leaf area over time measured using a digital camera and software (Fig. 1A, B). (B) Total root length over time measured using a flatbed scanner and software (Fig. 1C, D). (C) Projected leaf area from (A) plotted against total root length from (B) of whole plants over time. (D) Length of primary axile roots (measured as shown in Fig. 1C, D). (E) Length of lateral roots (total root length − primary axile root total length). (F) Root hair length on primary axile roots (measured as shown in Fig. 1E, F.) All data points are means ±standard error (*n*=10). Asterisks indicate statistically significant differences by unpaired *t*-test: *P<0.05, **P<0.01, ***P<0.001. Solid symbols refer to plants with bacteria treatment; black symbols indicate high N supply; red symbols indicate low N. Bacterial effects are shown with lines (5 mM versus 5 mM+HS, black line; and 0.5 mM versus 0.5 mM+HS, red line); for other comparisons see Supplementary Table S2; *n*=10, except 5 mM where *n*=9. DAT, days after transplanting to EcoFAB-N.

The shoot response to the N availability showed a larger PLA for 5 mM NH_4_NO_3_-grown plants than those grown at 0.5 mM (Fig. 3A). The difference between the non-inoculated plants in the 5 mM and 0.5 mM NH_4_NO_3_ media became quantifiable via the image analysis at 11 DAT and was maintained throughout the time series (Fig. 3A; Supplementary Table S2A). PLA increase in 5 mM NH_4_NO_3_ ranged from 32.0% (Student’s *t*-test; *P*<0.01) at 11 DAT, to 256.9% at 28 DAT (*t*-test; *P*<0.001), compared with shoots on 0.5 mM NH_4_NO_3_.

Shoot leaf area was enhanced by *H. seropedicae* at low N. At 5 mM, the difference in the leaf area was not significant between the inoculated and non-inoculated plants. However, at 0.5 mM NH_4_NO_3_, inoculated plants showed the first significant increase of PLA at 23 DAT (19.7%, *P*<0.05) over the non-inoculated plants. At 28 DAT the difference increased to 24.3%, indicating that a longer time series may record larger benefits of the inoculation. Shoot dry weight of inoculated 0.5 mM NH_4_NO_3_ plants was higher than the non-inoculated counterparts only at the start of the time series (11 DAT, *P*<0.05) (Supplementary Fig. S3C).

*Herbaspirillum seropedicae*-inoculated plants in both N conditions showed an increase in the total root length mainly explained by increase of the primary root length (Fig. 3B, D); in addition, inoculated plants had shorter root hairs (Fig. 3F). The magnitude and speed at which these changes appeared was influenced by the availability of N in the medium. The total root length was not different between plants grown in the two nitrogen conditions (Fig. 3B; Supplementary Table S2B). However, inoculation with *H. seropedicae* increased the total root length in both N conditions. At 5 mM NH_4_NO_3_ we only found longer roots in the inoculated plants at 28 DAT (34.0%; Fig 3B). Noticeably, in the 0.5 mM NH_4_NO_3_ conditions, faster root growth was observed among inoculated plants much sooner, by 11 DAT (29.1%), and this remained the case until 28 DAT when the inoculated roots were 25.5% longer than the non-inoculated group. Independent of inoculation, higher root allocation for a given leaf area was observed in 0.5 mM NH_4_NO_3_-grown plants, but higher shoot investment for a given root length was observed in 5 mM NH_4_NO_3_-grown plants (Fig. 3C).

Nitrogen supply altered primary roots (Fig. 3D; Supplementary Table S2C), shortening axiles when grown in 5 mM NH_4_NO_3_ by 19% (*P*<0.01) at 11 DAT and 13.7% (*P*<0.05) at 28 DAT, compared with 0.5 mM NH_4_NO_3_ at the same time point. In contrast, the primary root length was increased with *H. seropedicae* at both N levels (5 mM NH_4_NO_3_: 17.6% at 11 DAT and 30.1% at 28 DAT; 0.5 mM NH_4_NO_3_: 19% at 11 DAT (*P*<0.001) and 30.0% at 28 DAT (*P*<0.001)). Lateral root length was not influenced by *H. seropedicae* inoculation (Fig. 3E; Supplementary Table S2D).

*Brachypodium distachyon* had shorter root hairs at higher N availability and upon inoculation with *H. seropedicae*. Root hairs responded more to N availability in the medium than to the presence of *H. seropedicae*, with shorter root hairs at high N compared with low N. At 5 mM NH_4_NO_3_ root hair length was on average 23.5% shorter at 4 DAT, and the difference increased to 51.9% at 28 DAT, compared with root hairs of plants grown at 0.5 mM NH_4_NO_3_ (Fig. 3F; Supplementary Table S2E). Inoculation with *H. seropedicae* resulted in regulation of the root hair length, through time, for the plants in each condition. While *H. seropedicae* decreased root hair length in both N conditions (Fig. 3F), surprisingly the root hairs of inoculated plants at 5 mM NH_4_NO_3_ decreased first, with significant decrease measured from 15.4% (17 DAT) to 30.8% (28 DAT) compared with non-inoculated plants at the same condition. The decrease at 0.5 mM NH_4_NO_3_ was similar at 23 DAT (23.6%) and 28 DAT (25.9%), and the absolute decrease (in mm) was greater in the 0.5 mM NH_4_NO_3_ roots (Fig. 3F).

### N uptake and allocation dynamics

*Herbaspirillum seropedicae* influenced the *B. distachyon* N allocation between root and shoot at low N availability. Plants grown on 5 mM NH_4_NO_3_ had higher N content than plants grown on 0.5 mM, and also had more biomass (Supplementary Fig. S3A–D). When we compared the absolute N amount in shoots of inoculated plants compared with their non-inoculated counterparts, there was no significant difference (Supplementary Fig. S3A), and this was the case for roots as well (Supplementary Fig. S3B). However, expressing N as a percentage of weight showed that, in certain situations, inoculated plants can produce more biomass per unit N. This is obvious in shoots of inoculated plants at 5 mM NH_4_NO_3_ at 28 DAT (Supplementary Fig. S3C, E) and roots of inoculated plants at 0.5 NH_4_NO_3_ at 28 DAT (Supplementary Fig. S3D, F). This opens up questions about perturbations in N-metabolism enzymes and transporters, and root to shoot N-allocation mechanisms upon inoculation (addressed below). Indeed, looking at the shoot/root N ratios at 0.5 mM NH_4_NO_3_, inoculation resulted in a significantly higher shoot allocation at 11 and 20 DAT but not at 28 DAT (Supplementary Fig. S3G, H).

**Fig. 4.**
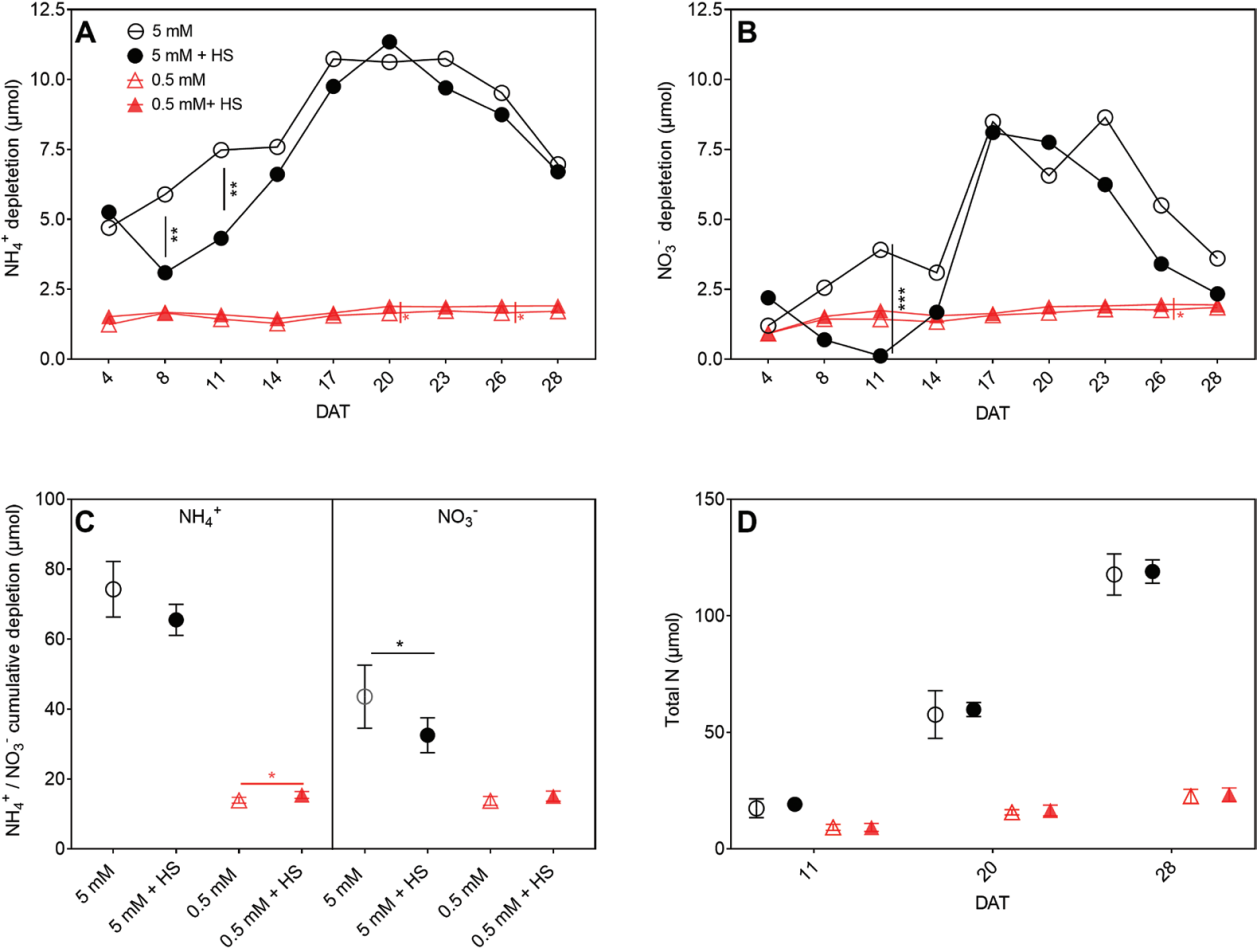
Nitrogen (NH_4_^+^, NO_3_^−^) depletion from EcoFAB-N medium over time and total N in *B. distachyon* grown in medium with 5 mM or 0.5 mM NH_4_NO_3_ and with or without root-associated bacteria of *H*. *seropedicae* (HS). (A) NH_4_^+^ depletion in EcoFAB medium was calculated as (initial medium NH_4_^+^ concentration at filling – final collected medium NH_4_^+^ concentration) × medium volume over time. NH_4_^+^ concentration was measured by continuous flow analysis and medium volume was measured by pipette. (B) NO_3_^−^ depletion was calculated as (initial medium NO_3_^−^ concentration at filling – final collected medium NO_3_^−^ concentration) × medium volume. NO_3_^−^ concentration were measured by ion chromatography and medium volume was measured by pipette. (C) Cumulative NH_4_^+^ or NO_3_^−^ depletion by *B. distachyon* after 28 DAT, calculated as sum of NH_4_^+^ (A) and NO_3_^−^ depletion (B) from 4 DAT to 28 DAT. (D) Total N per plant over time, measured by CHNS analyser. Points in (A–C) represent the mean of *n*=5 individual EcoFAB-N units each with one plant ±standard error. Points in (D) represent means ±standard error of *n*=3 EcoFAB-N units each with one plant at 11 and 20 DAT, and *n*=5 EcoFAB-N units each with one plant at 28 DAT. Asterisks indicate statistically significant difference by unpaired *t*-test: *P<0.05, **P<0.01, ***P<0.001. Bacterial effects (5 mM versus 5 mM+HS and 0.5 mM versus 0.5 mM+HS) are showed on the graphs; for other comparisons see Supplementary Table S3. DAT, days after transplanting into EcoFAB-N; HS, *H*. *seropedicae*.

The addition of *H. seropedicae* influenced uptake of N from the medium (in the form of NH_4_^+^ or NO_3_^−^ (Fig. 4A, B). As expected, non-inoculated plants grown in conditions in which there was more available N also managed to take up more than non-inoculated plants at low N (Fig. 4A, B). Compared with 0.5 mM NH_4_NO_3_, the NH_4_^+^ depletion from the medium at 5 mM NH_4_NO_3_ was 3.79-fold higher compared with 0.5 mM NH_4_NO_3_ at 4 DAT. The NH_4_^+^ depletion increased to 6.88-fold at 17 DAT and decreased to 4.08-fold at 28 DAT (Fig. 4A). Similarly, the NO_3_^−^ depletion at 5 mM NH_4_NO_3_ was 1.30-fold higher compared with 0.5 mM NH_4_NO_3_ at 4 DAT, and then it increased to 5.41-fold at 17 DAT and decreased to 1.95 at 28 DAT (Fig. 4B).

**Fig. 5.**
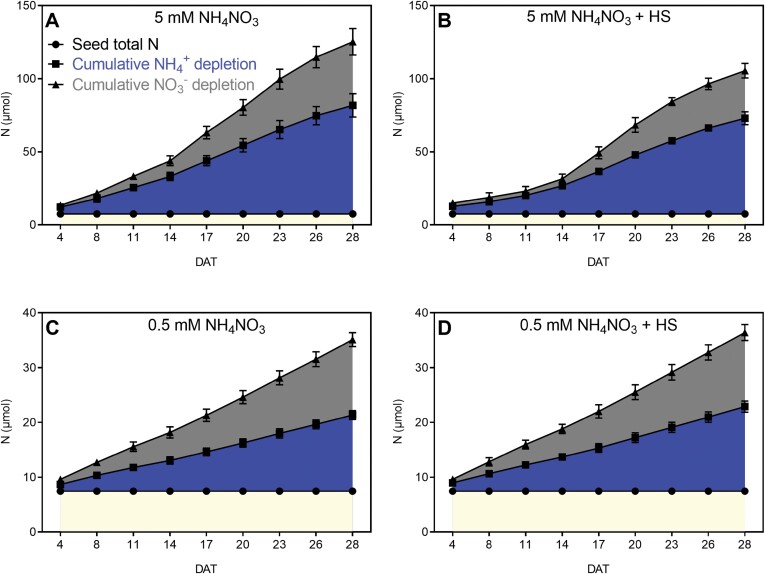
Cumulative N depletion over time from Eco-FAB-N medium by *B. distachyon* roots exposed to 5 mM NH_4_NO_3_ or 0.5 mM NH_4_NO_3_, with or without *H*. *seropedicae* (HS). For calculations see Eqs 3–6. Data are means ±standard error (*n*=5 EcoFAB-N units per treatment). (A) 5 mM NH_4_NO_3_; (B) 5 mM NH_4_NO_3_ with *H*. *seropedicae*; (C) 0.5 mM NH_4_NO_3_; (D) 0.5 mM NH_4_NO with *H*. *Seropedicae.* Note axes are different for N levels. N contained in seeds is marked in yellow, ammonium in dark blue; nitrate in gray. For statistical differences see Supplementary Table S4.

Inoculation with *H. seropedicae* results in slight alterations of NH_4_^+^ depletion in the medium. At 5 mM NH_4_NO_3_, the NH_4_^+^ depletion was significantly decreased at 8 DAT and 11 DAT. In 0.5 mM NH_4_NO_3_, on the contrary, the depletion of NH_4_^+^ was significantly increased at 20 DAT and 26 DAT. Thus, *H. seropedicae* differentially impacts the NH_4_^+^ uptake from the medium based on NH_4_^+^ availability and time from inoculation (Figs 4, 5). In the case of 5 mM NH_4_NO_3_, the NO_3_^−^ depletion was similarly decreased in inoculated EcoFABs at 11 DAT. The NO_3_^−^ depletion at 0.5 mM NH_4_NO_3_ was significantly increased at 26 DAT, at a similar time as NH_4_^+^ depletion increases in this condition. Thus, in inoculated EcoFAB-Ns we see coordinated regulation of NH_4_^+^ and NO_3_^−^ uptake, based on N availability. At the final time point (28 DAT) in the non-inoculated EcoFABs, the sum of the total depleted NH_4_^+^ from 5 mM NH_4_NO_3_ was 5.34-fold higher than the cumulatively depleted NH_4_^+^ from 0.5 mM NH_4_NO_3_. Inoculation with *H. seropedicae* only resulted in a significant change at 0.5 mM NH_4_NO_3_, where the cumulative depletion of NH_4_^+^ was significantly increased by 11.0% in the inoculated EcoFAB-Ns (Fig. 4C). Thus, in this case addition of *H. seropedicae* resulted in more NH_4_^+^ missing from the medium at 0.5 mM NH_4_NO_3_. The NO_3_^−^ cumulative depletion for non-inoculated plants (calculated at 28 DAT) at 5 mM NH_4_NO_3_ was 3.17-fold higher compared with NO_3_^−^ depletion in 0.5 mM NH_4_NO_3_. Interestingly, at 5 mM NH_4_NO_3_ after inoculation with *H. seropedicae* the cumulative depletion of NO_3_^−^ was significantly lower by 25.34% (Fig. 4C). Thus, *H. seropedicae* addition at 5 mM NH_4_NO_3_ resulted in less NO_3_^−^ missing from the medium.

### N mass balance of *B. distachyon* plants grown in EcoFAB-N with *H. seropedicae* suggests N fixation

**Fig. 6.**
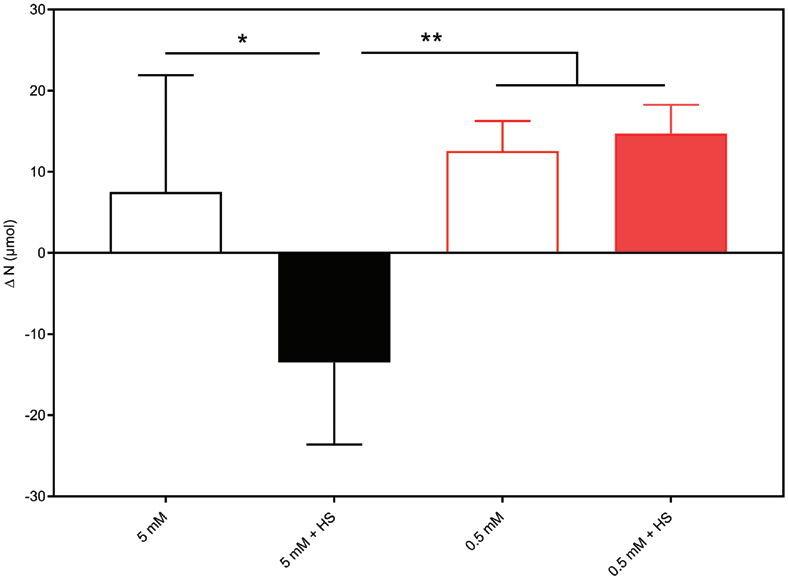
Difference between the total N depletion from the EcoFAB-N medium and N available in the seed, and the total plant N of *B. distachyon* after 28 DAT grown with 5 mM or 0.5 mM NH_4_NO_3_, with or without *H*. *seropedicae* (HS). For calculation see Eqs 3–7. Data are means ±standard error of five EcoFAB-N units per treatment. Red bars, low N; black bars, high N; solid bars, *H*. *Seropedicae* (HS). Asterisks indicate statistically significant differences by unpaired *t*-test: *P<0.05, **P<0.01 (Supplementary Table S4D).

To check if the plants contained more or less N than what we provided, we compared the cumulative depletion of N until 28 DAT, incorporating N available from the seed, as well as NO_3_^−^ and NH_4_^+^ missing from the medium, with the total N in *B. distachyon* at 28 DAT (Figs 4D, 5; Eq. 7). The ∆N, calculated as cumulative N depletion minus total N in plant, at 5 mM NH_4_NO_3_ was negative (Fig. 6). In this situation the plants contained an additional 13.49 µmol N, and it is tempting to speculate that this portion may have been provided by nitrogen fixation of *H. seropedicae*.

### Transcripts in roots and shoots of *B. distachyon* respond to *H. seropedicae*, N availability, and plant development

The observed changes in N uptake in the plant led us to ask if *B. distachyon* adjusts the expression of genes involved in central nitrogen metabolism, or if perhaps a hormone like auxin is regulating growth in the presence of *H. seropedicae* under the different nutrient levels and growth stages.

**Fig. 7.**
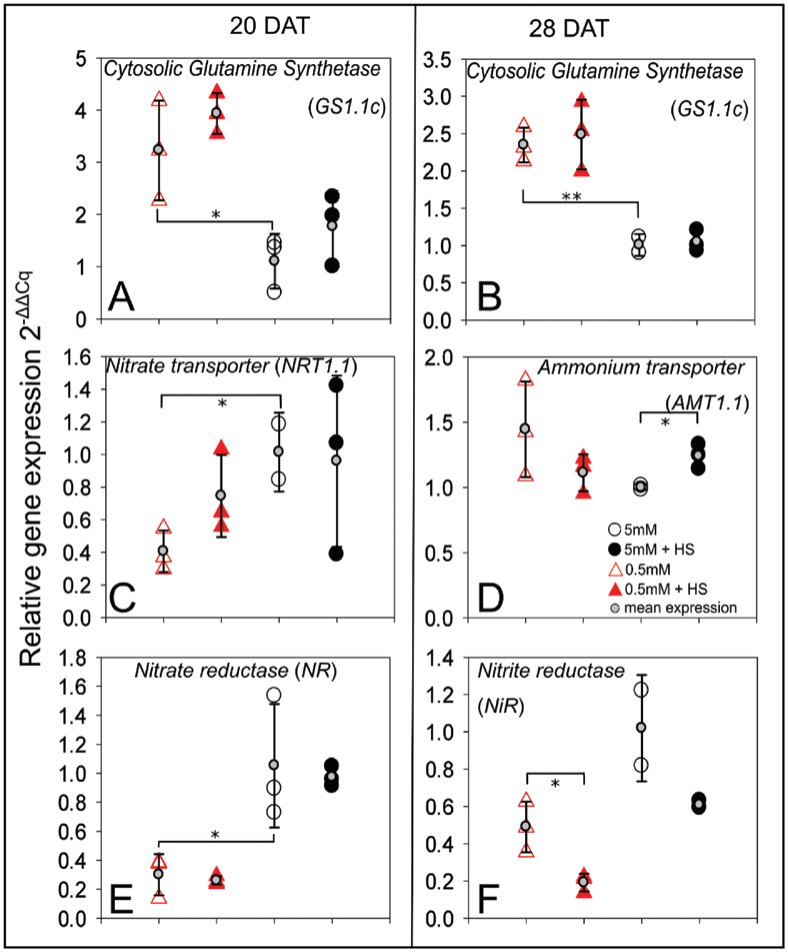
Quantitative real-time PCR of selected root transcripts. Relative expression level of *AMT1.1*, *NRT1.1*, *GS1.1c*, *NiR*, and *NR*, in *B. distachyon* roots grown in 5 mM NH_4_NO_3_ or 0.5 mM NH_4_NO_3_, with or without *H*. *seropedicae* (HS), in EcoFAB-N chambers for 20 and 28 DAT. (Additional time-points, without significant differences, are provided in Supplementary Fig. S4A.) Expression is normalized to *UBQ10*, and presented in comparison with the mean expression in 5 mM NH_4_NO_3_ at the respective time point. The expression of biological replicates is depicted using large circles or triangles, while the mean expression is shown on top of each condition using small gray circles and standard deviation error bars. A minimum of two biological replicates are shown. Asterisks indicate statistical differences by unpaired *t*-test for three comparisons: 0.5 versus 5 mM NH_4_NO_3_ and inoculated versus non-inoculated at each respective NH_4_NO_3_ level; and statistical differences by pairwise *t*-test for three comparisons: 0.5 versus 5 mM NH_4_NO_3_ and inoculated versus non-inoculated at each respective NH_4_NO_3_ level; *P<0.05, **P<0.01. *y*-Axis: relative expression levels (); black open circles, 5 mM NH_4_NO_3_; black filled circles, 5 mM NH_4_NO_3_ inoculated with *H*. *seropedicae* (HS); red open triangles, 0.5 mM NH_4_NO_3_; red filled triangles, 0.5 mM NH_4_NO_3_ inoculated with *H*. *seropedicae* (HS).

**Fig. 8.**
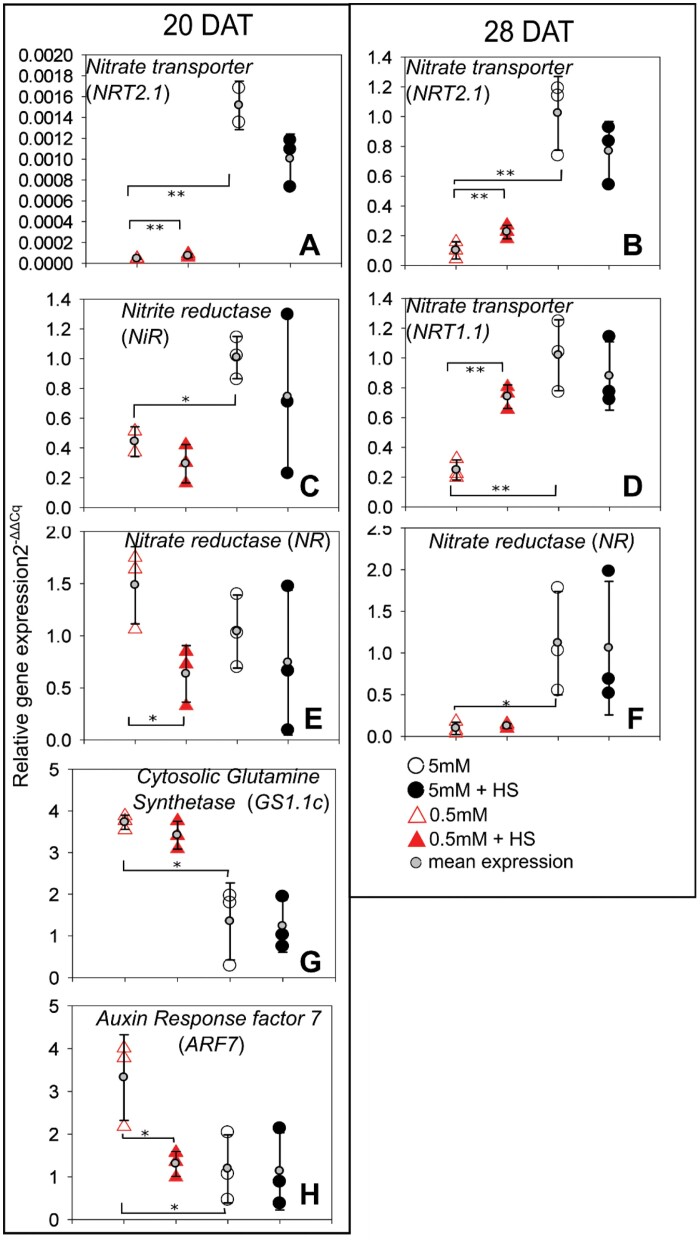
Quantitative real-time PCR of selected shoot transcripts. Relative expression levels () of *NRT1.1*, *NRT2.1*, *GS1.1c*, *NiR*, *NR*, and *ARF7* in *B. distachyon* shoots grown in 5 mM NH_4_NO_3_ or 0.5 mM NH_4_NO_3_ with or without *H*. *seropedicae* (HS), in EcoFAB chambers at 20 and 28 DAT. (Additional time-points, without significant differences provided in Supplementary Fig. S4B.) Expression is normalized to *UBQ10*, and presented in comparison with the mean expression in 5 mM NH_4_NO_3_, which is set to 1, at each respective time point. The expression of biological replicates is depicted using large circles or triangles and the mean expression is shown on top of the biological replicates using small gray circles and standard deviation error bars. A minimum of two biological replicates are shown. Asterisks indicate statistical differences by unpaired *t*-test: for three comparisons: 0.5 versus 5 mM NH_4_NO_3_ and inoculated versus non-inoculated at each respective NH_4_NO_3_ level; *P<0.05, **P<0.01. *y*-Axis: relative expression levels (), black open circles, 5 mM NH_4_NO_3_; black filled circles, 5 mM NH_4_NO_3_ inoculated with *H*. *seropedicae* (HS); red open triangles, 0.5 mM NH_4_NO_3_; red filled triangles, 0.5 mM NH_4_NO_3_ inoculated with *H*. *seropedicae* (HS).

Comparative transcriptional analysis was performed on *B. distachyon* to determine how the following impacted expression levels: (i) N concentration, observed as the comparison of the non-inoculated plants at 0.5 and 5 mM NH_4_NO_3_; (ii) *H. seropedicae* at a given N level; and (iii) the time point in (i) and (ii) (i.e. will the same transcript behavior be observed at both time points) (Figs 7, 8).

Transcriptional comparison at different N concentrations found firstly that for roots, in all but one case, expression of the genes for the enzymes of central N metabolism, glutamine synthetase, nitrate reductase, and nitrite reductase, was correlated with N availability at both time points (Fig. 7A, B, E, F; Supplementary Fig. S4A). However, transcription of the transporter genes varied more between biological replicates, so a consistent trend was not always observed (Supplementary Fig. S4A). In shoots, expression of genes for enzymes from central N metabolism (in non-inoculated plants) showed changes based on N supply only at some time points (20 DAT: *Cytosolic Glutamine Synthetase* (*GS1.1c*) and *Nitrite Reductase* (*NiR*), Fig. 8C, G; 28 DAT: *Nitrate Reductase* (*NR*), Fig. 8F). There was an opposite behavior between *GS1.1*, where the low N plants expressed the *GS* isoform at a higher level than the 5 mM plants, and *NiR* and *NR*, where the 0.5 mM plants expressed the genes for nitrate reduction enzymes at lower levels than the 5 mM plants. Interestingly this was also reflected in the behavior of the nitrate transporter genes *NRT1.1* at 28 DAT and *NRT2.1* at 20 and 28 DAT (Fig. 8A, B, D).

Secondly, *H. seropedicae* influenced the expression in both roots and shoots, albeit to a different extent. In roots, *H. seropedicae* increased the expression of the ammonium transporter gene *AMT1.1* in 5 mM NH_4_NO_3_ at 28 DAT compared with the non-inoculated plants (Fig. 7D). On the other hand, *H. seropedicae* decreased the expression of *NiR* in roots grown at 0.5 mM at 28 DAT (Fig. 7F). In shoots, *H. seropedicae* increased the expression of *NRT1.1* at 0.5 mM at 28 DAT, as well as *NRT2.1* at both time points (Fig. 8A, B, D). However, *H. seropedicae* decreased the expression of both *NR* and *Auxin Response factor 7* (*ARF7*) at 0.5 mM at 20 DAT (Fig. 8E, H).

Thirdly, time, or rather developmental point, also played a role in transcript expression, especially since plants might still be relying on seed reserves. Thus, if we compare the behavior of non-inoculated roots at 20 and 28 DAT, there were differences for *NRT1.1* and *NR* (Fig. 7C, E; Supplementary Fig. S4A). In shoots, the expression of non-inoculated transcripts changed through time for *NRT1.1*, *ARF7*, and *GS1.1c* (Fig. 7D, G, H; Supplementary Fig. S4B). Similarly, the time that *H. seropedicae* interacts with the plant also plays a role in the expression. Thus, in roots there was increased *AMT1.1* expression for 5 mM plants at 28 DAT (Fig. 7D) and decreased *NiR* expression in 0.5 mM plants at 28 DAT (Fig. 7F). In shoots, many of the transcripts related to nitrate uptake and reduction at 0.5 mM were affected by the presence of *H. seropedicae*, but *NRT1.1* was increased at 28 DAT only, whereas *NRT2.1* was increased at both time points (Fig. 8A, B, D). On the other hand, *NR* was decreased at 20 DAT only (Fig. 8E). *ARF7* was also repressed only at 20 DAT, in 0.5 mM plants (Fig. 8H).

## Discussion

**Fig 9.**
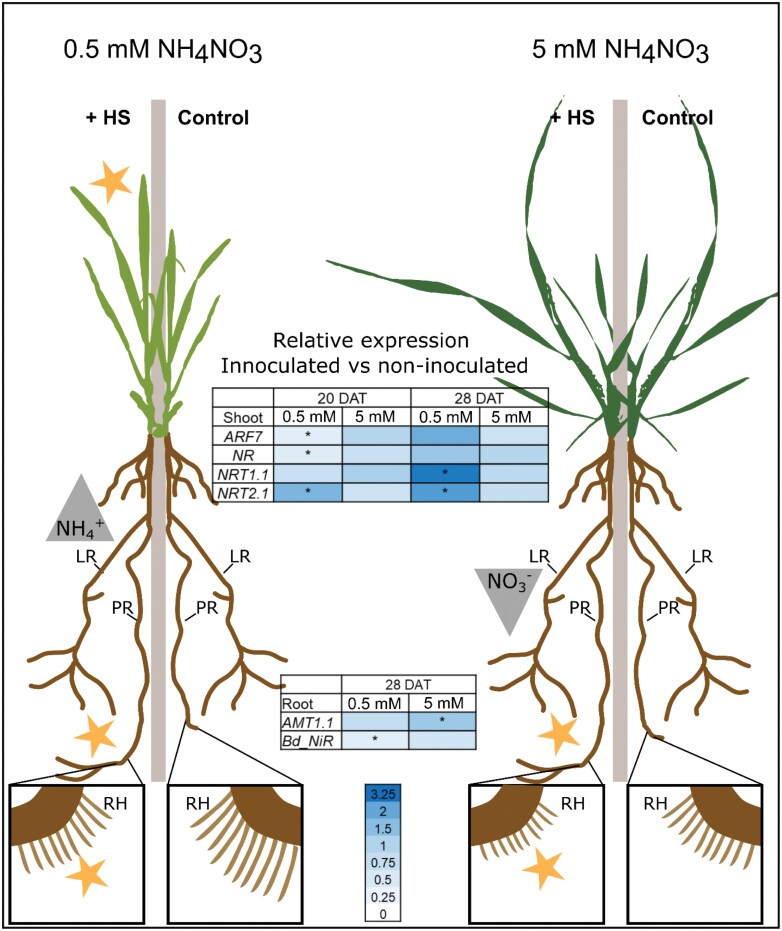
Summary of the response of *B. distachyon* to inoculation with *H. seropedicae* (HS) in two nitrogen conditions. At 0.5 mM NH_4_NO_3_ HS causes increase of leaf area, elongation of primary root, and shortening of root hairs. Inoculated plants also depleted NH_4_^+^ faster from the medium. Their nitrate uptake genes *NRT2.1 (both time points)* and *NRT1.1 (28 DAT)* in shoot have higher expression, corresponding to behavior at low NO_3_^−^. At 5 mM NH_4_NO_3_ inoculated plants show longer primary roots and shorter root hairs than non-inoculated plants. They deplete less NO_3_^−^ from the medium. Yellow stars indicate significant differences by non-invasive phenotyping. Relative expression, as ratio of inoculated versus non-inoculated is shown in the heat maps, with high (dark blue) and low ratio (white); asterisks indicate significant *t*-test in the inoculated versus non-inoculated plants. N-form uptake (gray triangles): increased depletion from medium (upright traingle), decreased depletion (inverted triangle). Control, non-inoculated; +HS, inoculated plants; LR, lateral roots; PR, primary root; RH, root hairs.

The EcoFAB-N coupled to whole plant imaging and N mass-balance analysis revealed a dynamic *H. seropedicae*–*B. distachyon* phenotype. N availability and time were the most important variables, and root and leaf responses varied in magnitude and direction. Further, inoculation promoted primary root growth, but inhibited root hair length, irrespective of nitrogen availability and time, while nitrogen form (nitrate or ammonium) and potentially N source (medium or bacterial) also varied. The main findings of the study are summarized in Fig. 9. Dynamics are important to consider when interpreting variable responses to N-fixing bacteria in cereals in agriculture.

### EcoFAB-N and the non-invasive phenotyping pipeline are flexible and allow further expansion by the scientific community

One big advantage of the open source EcoFAB platform is that the chamber is adjustable and can therefore be configured based on the experimental needs, e.g. nutrition and aeration in the case of aerobic bacterial interactions (this work, EcoFAB-N), or plant space requirements (Gao *et al.*, 2018). We show here that the closed EcoFAB-N system provides an accurate method to study mass balance, such as N mass balance investigated in this study, which is challenging in natural systems. Mass balance analyses coupled to time-resolved phenotypic data are a useful way to estimate nutrients taken up by plants growing with complex biotic and abiotic nutrient forms (Mau *et al.*, 2021). By extending the EcoFAB platform to include ­non-invasive shoot and root imaging, we increased the throughput of growth phenotyping to a turnover of 25–30 plants h^−1^. The EcoFAB-N pipeline allows study of the whole plant growth and N fluxes while maintaining the gnotobiotic environment, with integration of molecular responses within the dynamic phenotype at specific time points, via invasive harvests.

We found that *B. distachyon* grown in high N medium had greater leaf area and shoot biomass, and comparable root length to *B. distachyon* grown at lower N. This fits with expected plant behavior under N limitation (Cambui *et al.*, 2011; David *et al.*, 2019). The resource allocation in the case of the high 5 mM NH_4_NO_3_ was greater towards shoots, compared with in the case of 0.5 mM NH_4_NO_3_, which was directed to the root system (Fig. 3C). The results from the EcoFAB-N also aligned well to wheat responses to similar N levels when grown in paper pouches (Tolley and Mohammadi, 2020).

Additionally, we observed a root hair response that was linked to N supply. Here we observe shorter root hairs at 5 mM NH_4_NO_3_ than at 0.5 mM NH_4_NO_3_, although newly produced root hairs were shortening through time. While root hairs have been shown to respond to P deficiency and influence uptake, information about N is not as clear. However, in tomato, certain ammonium and nitrate transporters are expressed in the root hairs, suggesting root hair cells may have a role in N uptake (Gilroy and Jones, 2000). Studies of wild grasses reported shorter root hairs with increased N availability (Boot and Mensink, 1990). Recently a modeling approach indicated that root hairs are important during N deficiency (Saengwilai *et al.*, 2021). We conclude that root hair length is a trait worthy of further investigation for more N-efficient varieties, and that plant development is an important variable for full understanding of the contribution of length.

### The phenotypic and transcript responses of *B. distachyon* to *H. seropedicae* differed depending on the nutritional conditions and time

Our results lead us to the conclusion that *B. distachyon* responses to *H. seropedicae* inoculation are strongly dependent on the nutrient condition where the interaction takes place. We observed two types of response in inoculated plants. Firstly, conserved responses to *H. seropedicae* inoculation, like increase of total root length or decrease of root hair length, irrespective of the available N (Fig. 3B, F). The differences here can be described as a cumulative response to N availability and *H. seropedicae* inoculation. The second type of response was an N-related response to *H. seropedicae* in inoculated plants. For example, *H. seropedicae*-induced change in leaf area was increased only in the low N condition (Fig. 3A). Closer observation showed that several measured parameters fit into this group by showing different response to *H. seropedicae* inoculation in 5 mM and 0.5 mM NH_4_NO_3_ (i.e. leaf area, the depletion of NH_4_^+^ and NO_3_^−^, as well as expression of *AMT1.1* and *NiR* at 28 DAT, Figs 3A, 4C, 7D, F).

Time, both as a factor in plant development and from the moment of inoculation, also played a role in plant–bacterial dynamics, in addition to nutrient supply, consistent with Schillaci *et al.*, (2021). For example, at 11 DAT, total root length (Fig. 3B) increased in inoculated plants at 0.5 mM NH_4_NO_3_, but at 28 DAT total root length increase was only measured in the 5 mM NH_4_NO_3_ treatment.

We observed differences in the NH_4_^+^ and NO_3_^−^ depletion at different times as well, in inoculated and non-inoculated plants, and in both conditions (Fig. 4A, B), with decreased NH_4_^+^ and NO_3_^−^ depletion (lower uptake by the plant) in the 5 mM condition at the start of the time series (8 and 11 DAT) and slightly increased depletion (higher uptake) in the 0.5 mM condition towards the end of the time series. The molecular components in the N uptake system also varied through time (Figs 7, 8). The expected behavior in fully developed plants is that the *AMT* and *NRT* transporter genes, as well as enzymes, are more highly expressed at lower NH_4_^+^ and NO_3_^−^ concentrations (David *et al.*, 2019). While we often saw this behavior (e.g. *GS*, Figs 7A, B, 8G), we saw deviations as well. Notably most studies investigate plant responses at a specific time in development, but plant development including utilization of seed resources needs to be considered for proper understanding of the dynamic transcript expression (Enns *et al.*, 2006). We speculate that part of the N-related response was related to resources in the seed, and the amount impacts the expression of the various transcripts measured in this study. Particularly in roots, we saw a large variation among the monitored nitrogen transporters in this study, whereas the behavior of the central metabolic enzymes appeared to be more stable. A longer time series may lead to a better quantification of molecular responses in the future studies, but the data presented here already show similar *NR* (at 20 and 28 DAT; Fig. 7E; Supplementary Fig. S4A) and *NiR* (28 DAT; Fig. 7F) expression. In inoculated plants, *NiR*, whose protein reduces NO_3_^−^ to NH_4_^+^, had lower transcript expression at 28 DAT in the 0.5 mM condition (Fig. 7F). The preceding enzyme in the NO_3_^−^ reduction pathway responded similarly (*NR*; Supplementary Fig. S4A) to *H. seropedicae* indicating that this is a robust response to the bacterium. The general expression pattern (lower expression at low N, higher in high N) was also followed by the nitrate transporter gene *NRT2.1*.

Interestingly the expression of the cytosolic glutamine synthetase gene (*GC1.1c*; Fig 7A, B), involved in NH_4_^+^ metabolism, in *B. distachyon* was not altered by *H. seropedicae*; and transcript expression pattern was opposite to the nitrate-related enzymes (higher expression in low N conditions compared with high N). This leads to two questions: does *B. distachyon* have a preference for ammonium rather than nitrate? Do specific transcripts respond to the presence of a given bacterial inoculant? Future EcoFAB-N experiments with KNO_3_ and (NH_4_)_2_SO_4_ as sole nitrogen sources combined with phenotyping and extensive sequencing will be needed to answer these questions.

### *H. seropedicae* inoculation altered N mass balance of *B. distachyon* in EcoFAB-N

Although N-fixation by free-living bacteria associated with grasses provides a lower amount of N to crops compared with nitrogen provided from *Rhizobium* spp. to leguminous plants (Rosenblueth *et al.*, 2018), a small contribution to grasses is important owing to the large global cereal agriculture. In this study, *B. distachyon* plants grown for 28 DAT supplied at 5 mM NH_4_NO_3_ contained 11.34% N that could not be accounted for based on medium uptake (calculated as in Fig 6, black bar as percentage of total plant N at 28 DAT, Supplementary Fig. S3). The current working hypothesis is that this was provided through the activity of *H. seropedicae.* Interestingly we did not find any obvious contribution of *H. seropedicae* to the N content in plants grown on 0.5 mM NH_4_NO_3_. Future experiments using *H. seropedicae* mutant strains without N-fixation genes, or a change of the ^15^N composition in the plant, could be used to confirm the contribution of the bacterium through N fixation. Bacterial gene expression studies at the two conditions could further clarify the bacterial variability and adjustment to the interaction at various N levels.

Besides the nitrogen fixation, *H. seropedicae* might also stimulate N uptake from soil/medium, which has not been intensively investigated. Ribaudo *et al.* (2006) reported that *H. seropedicae* inoculation of maize positively affected activities of ammonium assimilation enzymes (Ribaudo *et al.*, 2006). In this study, we observed increased ammonium and nitrate depletion only in the medium at 0.5 mM NH_4_NO_3_. We thus hypothesize that the rapid N depletion under low N conditions may be stimulated by *H. seropedicae*.

Looking forward, this study stimulates plant-specific questions: Does *B. distachyon* have a preferred N-form? Do root hairs express transporters for N uptake and could this knowledge be exploited for plants with improved N uptake? Finally, what is the best time for bacterial inoculation in the context of plant developmental stage, and which environmental prerequisites exist for a beneficial plant–microbe interaction in the field?

### Conclusion

Improvement of plant performance through the use of beneficial microbes is dynamic and thus must include understanding of the interaction through the plant’s developmental period under varying abiotic conditions including fertilizer supply.

The adaptations to the gnotobiotic EcoFAB-N chamber in this study allow its application to nitrogen-related experiments with the use of N-fixing bacteria, and can be expanded to other nutrient conditions. The non-invasive phenotyping pipeline allows precise monitoring of root and shoot parameters through time, including analysis of primary and lateral root lengths and root hairs, which are not possible using simple end-point measurements. Finally, the approach for the mass-balance calculation is transferable to multiple nutrients and potentially beneficial organisms including P from algal biomass (Mau *et al.*, 2021). In this particular case we saw promotive action by *H. seropedicae* on *B. distachyon* growth at high and low NH_4_NO_3_. The time-resolved phenotypic and molecular data, however, point to distinct modes of action: at 5 mM the benefit appears through N fixation, while at 0.5 mM the mechanism appears to be plant physiological, with *H. seropedicae* promoting uptake of N from the root medium.

## Supplementary data

The following supplementary data are available at *JXB* online.

Fig. S1. Pilot study of ammonium depletion in original EcoFABs to establish the size and design of EcoFAB-N.

Fig. S2. Non-invasive *B. distachyon* leaf area and invasive leaf area or shoot fresh weight correlations.

Fig. S3. *Brachypodium distachyon* shoot and root total N, dry weight, and N concentration.

Fig. S4. Quantitative real-time PCR of selected root and shoot transcripts at 20 and 28 DAT.

Table S1. Primers used in this study.

Table S2. Growth of *B. distachyon* measured non-destructively over time when roots exposed to 5 mM NH_4_NO_3_ or 0.5 mM NH_4_NO_3_, with or without *H. seropedicae* (HS), in EcoFAB-N chambers shown in Fig. 3.

Table S3. Ammonium and nitrate depletion in the EcoFAB medium, shown in Fig. 4A, B.

Table S4. Cumulative N depletion in the EcoFAB medium, shown in Fig. 5.

erac184_suppl_Supplementary_Figures_S1-S4_Tables_S1-S4

## Data Availability

The protocol for design and preparation of EcoFAB-N protocol is available under: dx.doi.org/10.17504/protocols.io.b53tq8nn. The protocol for non-invasive imaging of leaf area using a mobile phone in the EcoFAB-N is available under: dx.doi.org/10.17504/protocols.io.b53kq8kw. The protocol for live imaging of bacterial colonization of the plant root is available under: dx.doi.org/10.17504/protocols.io.b53uq8nw.
