# Supplementary material for: N-dependent dynamics of root growth and nitrate and ammonium uptake are altered by the bacterium Herbaspirillum seropedicae in the cereal model Brachypodium distachyon
Source: J Exp Bot. 2022 May 5;73(15):5306–21. doi: 10.1093/jxb/erac184 (PMC9440436; doi:10.1093/jxb/erac184)
Supplement: erac184_suppl_Supplementary_Figures_S1-S4_Tables_S1-S4 [file erac184_suppl_supplementary_figures_s1-s4_tables_s1-s4.pdf]

Kuang *et al.*, Supplemental Figures and Tables.

Supplemental Figures:

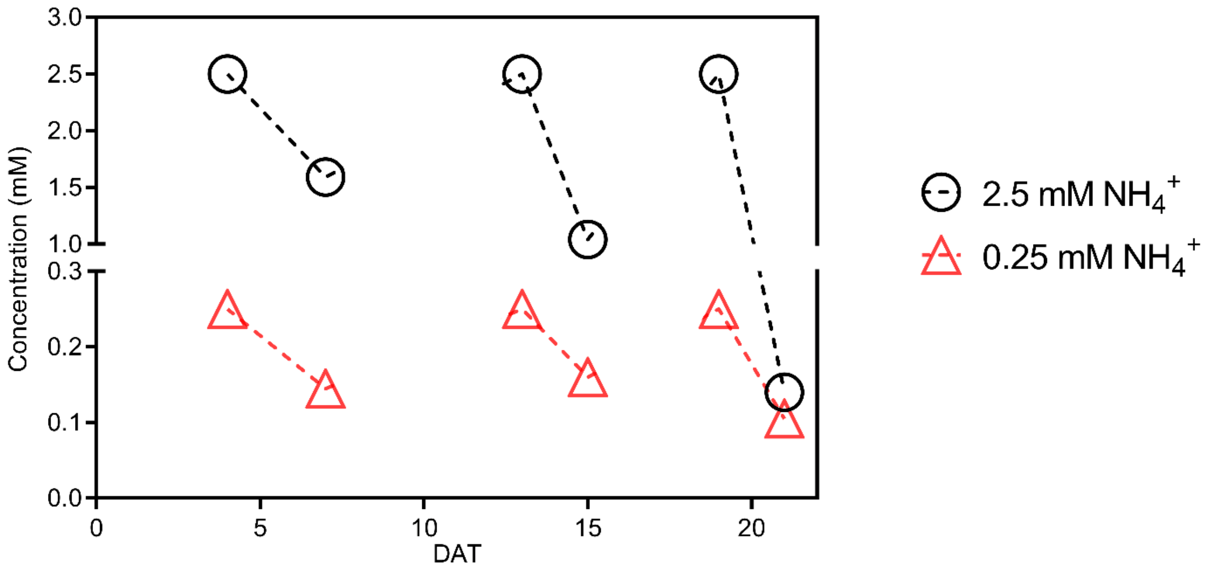

Fig. S1 Pilot study of ammonium depletion in original EcoFABs (Sasse et al., 2019) to establish the size and design of EcoFAB-N used here (see doi: dx.doi.org/10.17504/protocols.io.b53tq8nn). The EcoFAB had one *B. distachyon* plant growing in revised 0.5 MS medium with 2.5 mM NH_4NO_3 (2.5 mM NH_4) or 0.25 mM NH_4NO_3 (0.25 mM NH_4^+) for 21 days. Medium were changed at 4, 7 days after transplanting (DAT), followed with changing every 3 days until harvest at 21 DAT. Ammonium concentration in fill medium at 4, 13, 19 DAT and collect medium at 7, 15, 21 DAT was measured by ammonia assay kit (Sigma, AA0100). Data is one replicate.

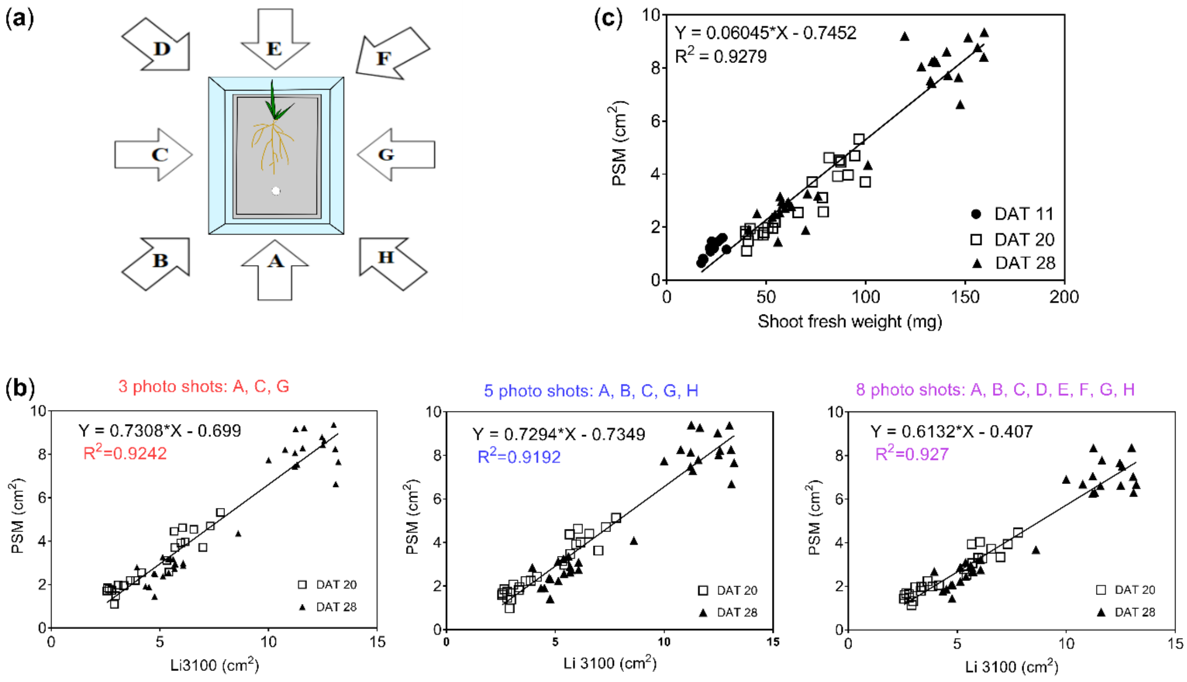

Fig. S2. Non-invasive *B. distachyon* leaf area and invasive leaf area or shoot fresh weight correlations. (a) Imaging scheme of the EcoFAB-N, using a mobile camera positioned at a 45° angle facing downwards. *B. distachyon* images were obtained with the Plant screen mobile software (PSM), and projected leaf area was calculated using the screen segmentation software (see doi: dx.doi.org/10.17504/protocols.io.b53kq8kw). (b) Optimization of number of shots required. Photos from 3, 5, 8 directions were taken at 20, 28 DAT and measured by the method showed in Fig. 1a, b. (c) Correlation between non-invasive leaf area obtained with 3 PSM images as shown in Fig. 1a, b and destructive shoot fresh weight measurements 11, 20, 28 DAT. N plants imaged: 11 DAT (n= 3); 20 DAT (n=6), 28 DAT (n=8).

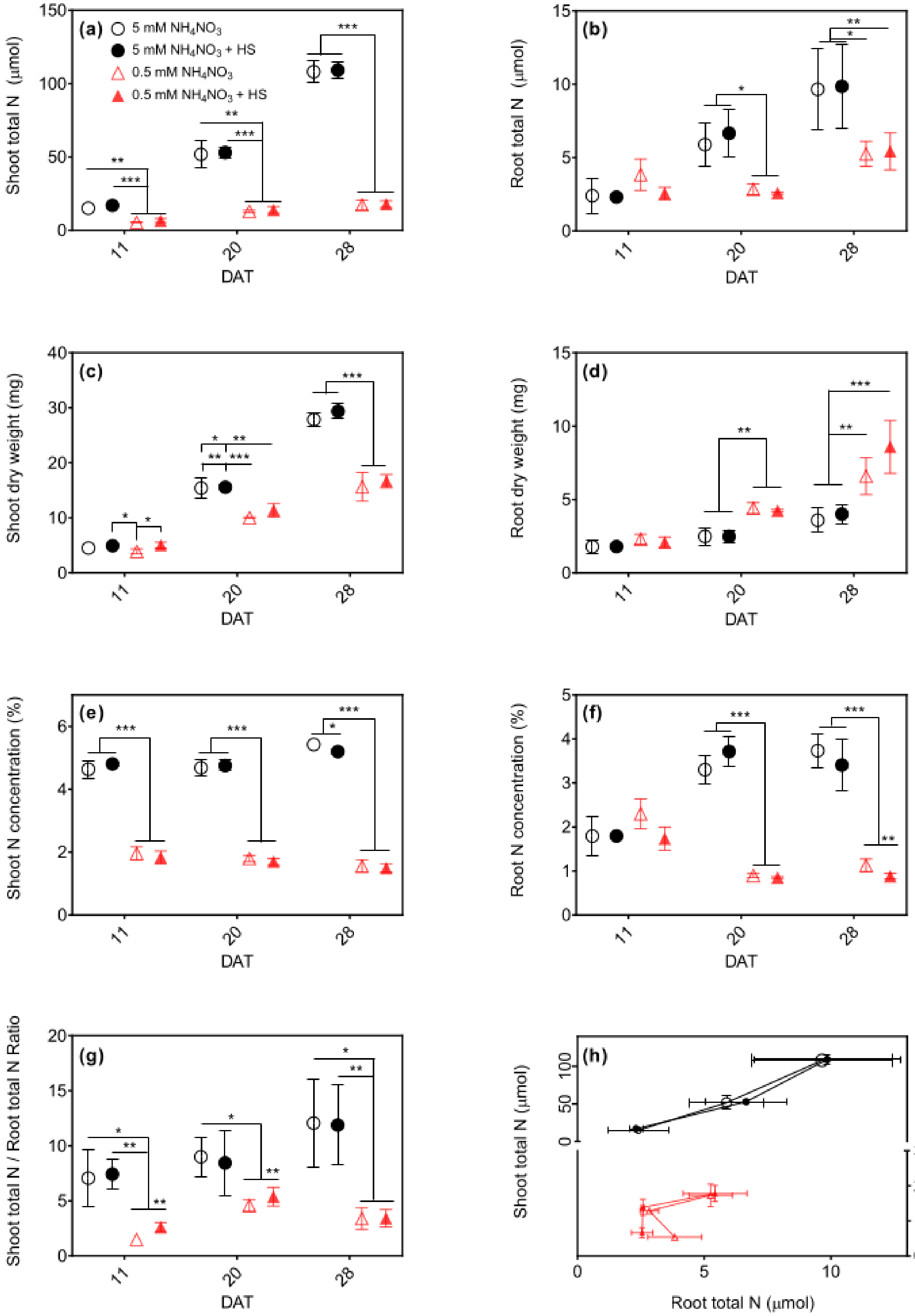
(a)
5 mM NH4NO3
5 mM NH4NO3 + HS
0.5 mM NH4NO3
0.5 mM NH4NO3 + HS
Shoot total N (μmol)
DAT
(b)
Root total N (μmol)
(c)
Shoot dry weight (mg)
(d)
Root dry weight (mg)
(e)
Shoot N concentration (%)
(f)
Root N concentration (%)
(g)
Shoot total N / Root total N Ratio
(h)
Shoot total N (μmol)
Root total N (μmol)

Fig.S3 *B. distachyon* shoot and root total N, dry weight, and N concentration. *B. distachyon* was grown in 5 mM NH_4NO_3 or 0.5 mM NH_4NO_3, with or without *H. seropedicae* (HS), in EcoFAB-N chambers. Data are means ± standard error (n = 3 at 11, 20 DAT and n = 5 at 28 DAT). (a) Shoot total N; (b) Root total N; (c) Shoot dry weight; (d) Root dry weight; (e) Shoot N concentration; (f) Root N concentration; (g) Shoot total N / Root total N ratio. (h) Allocation of total shoot total N (a) and root total N (b) of whole plants over time. Asterisk indicate statistically differences according to unpaired t-test at the 0.05 level. *, $p < 0.05$; **, $p < 0.01$; ***, $p < 0.001$.

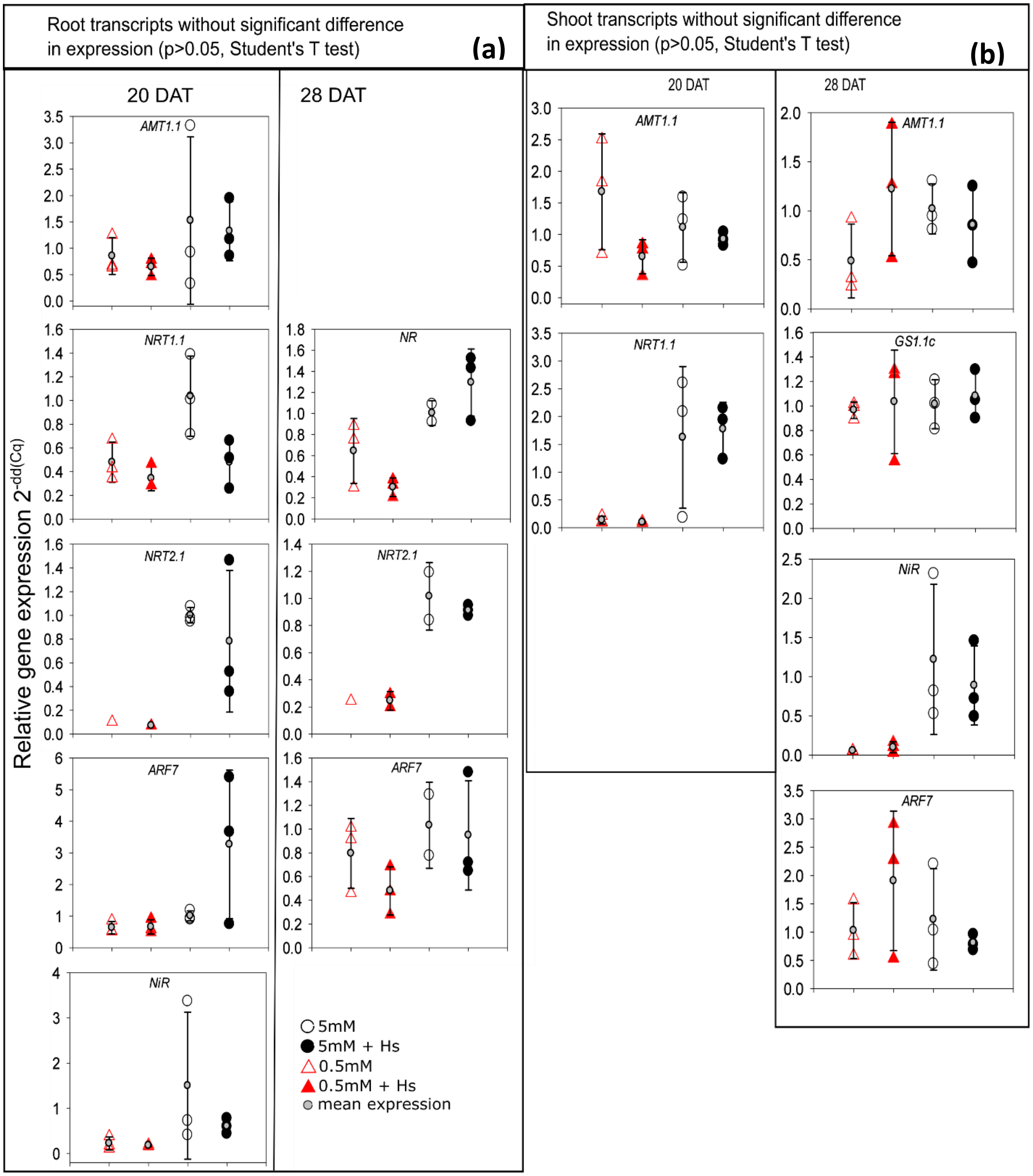

Fig S4: Quantitative Real-Time PCR of selected root (a) and shoot transcripts (b) at 20 and 28 DAT. Relative expression level of AMT1.1, NRT1.1, NRT2.1, NiR, NR, GS1.1c and ARF7 in *B. distachyon* roots, which were grown in 5 mM NH_4NO_3 or 0.5 mM NH_4NO_3, with or without *H. seropedicae* (HS), in EcoFAB-N chambers for 20, 28 DAT. Expression are normalized to UBQ10, and standardized to the expression in 5 mM NH_4NO_3 at the respective time point. The expression of biological replicates is

depicted using big circles or triangles, while the mean expression is shown on top of each condition using small gray circles and standard deviation error bars. A minimum of 2 biological replicates are shown, except: in NRT 2.1 in Root (20DAT, 0.5 NH_4NO_3 both conditions, and 28 DAT 0.5mM, non-inoculated) where n=1. Asterisks indicate statistical differences according to unpaired t-test for three comparisons: 0.5 vs 5mM NH_4NO_3, and inoculated vs non-inoculated at each respective NH_4NO_3 level. Ammonium transporter 1.1. (AMT1.1), Nitrate transporter 1.1 (NRT1.1), Nitrate transporter 2.1 (NRT2.1), Nitrite reductase (NiR), Nitrate reductase (NR), Cytosolic Glutamine Synthetase (GS1.1c) and Auxin response factor 7 (ARF7)

Supplemental tables

Table S1 Primers used in this study

Gene	Phytozome Gene Identifier	Primer	Sequence 5´ to 3´	NCBI Reference Sequence
Bd_UBQ10	BdiBd21-3.1G0441800	Fwd	TGGACTTGCTTCTGTCTGGG	NM_001317880
		Rev	ACAGGCATAACACTGACGAC	
Bd_SamDC	BdiBd21-3.5G0187900	Fwd	GATGGCGAGAACGTGGAGAA	XM_010241769.2
		Rev	AAACAAATTGCACGGGGACG	
Bd_NRT1.1A	BdiBd21-3.3G0226900	Fwd	GTGAAATGGGGAAGGGTTTT	XM_003573432.3
		Rev	ATGCAGCGACATTCAGTCAC	
Bd_NRT2.1	BdiBd21-3.2G0521300	Fwd	GGCAGCTCGACTTCTTCATC	XM_003572502.2
		Rev	TAGTCTAGGCGGCTGTGGTT	
Bd_GS1.1c	BdiBd21-3.3G0789600	Fwd	AGCACTCCCAAAAGCTCAAA	XM_010237849.3
		Rev	TGTAAATCGCCCAAATCCTC	
Bd_AMT1.1	BdiBd21-3.5G0198500	Fwd	AATCCCCAGATTCCCAAAAC	XM_003580067.3
		Rev	GTGAATCCTTCGTGGTTGCT	
Bd_NiR1	BdiBd21-3.3G0767200	Fwd	CAGGAGAAGGTGAAGCTGGG	XM_003570520.3
		Rev	GCAGCTTCAGACGCATCATG	
Bd_ARF7	BdiBd21-3.3G0608800	Fwd	GGAGGCATGCTGAAGAGTGT	XM_010237246.3 (Zhou *et al*, 2018)
		Rev	CAGCCTCCTTACACCGACTC	
Bd_NR1	BdiBd21-3.3G0500200	Fwd	GTCAAGCGCATCATCGTCAC	XM_003574559.3
		Rev	GTCGTGAATGCGTTGATGGG	
Bd_NRT2.5	BdiBd21-3.2G0609200	Fwd	GTCCAAGGCCAAGTTCAGGA	https://doi.org/10.1111/ppl.12716 (Wang, Hüner, & Tian, 2019).
		Rev	CCCAGATTGTCCCGGATGAG	
Hs_16s	1847008 [GenBank]	Fwd	TTCCCGGGTCTTGTACACAC	NR_029329.1
		Rev	CGTGCGCACTCTAGAAAGGA	
Universal bacterial primer	16s rRNA	27F	AGAGTTTGATCMTGGCTCAG	https://doi.org/10.1128/AEM.02272-07 (Frank *et al*., 2008)
		1492R	GGTTACCTTGTTACGACTT	

Table S2. Growth of *B. distachyon* measured non-destructively over time at 4, 11, 17, 23 and 28 days after transplanting (DAT) when roots exposed to 5 mM NH_4NO_3 or 0.5 mM NH_4NO_3, with or without *H. seropedicae* (HS), in EcoFAB-N chambers shown in Fig. 3 (n=10, except 5mM where n=9). All data through time with means ± standard error (n = 10); Asterisk indicate statistically differences according to unpaired t-test at the 0.05 level. ns, no significant; *, p < 0.05; **, p < 0.01; ***, p < 0.001; ***. (a) Project leaf area results; (b) Total root length; (c) Primary root total length; (d) Lateral root total length; (e) Root hair length.

(a) Project leaf area results

	Treatments	DAT 4	DAT 11	DAT 17	DAT 23	DAT 28
Project leaf area	5 mM	0.49 ± 0.10	1.29 ± 0.21	2.68 ± 0.49	6.20 ± 0.67	8.53 ± 0.59
(cm^2)	5 mM + HS	0.50 ± 0.12	1.35 ± 0.24	3.08 ± 0.39	5.92 ± 0.98	7.57 ± 1.37
	0.5 mM	0.47 ± 0.13	0.97 ± 0.17	1.39 ± 0.29	1.88 ± 0.36	2.39 ± 0.45
	0.5 mM + HS	0.50 ± 0.09	1.09 ± 0.15	1.63 ± 0.28	2.25 ± 0.16	2.97 ± 0.29
T test						
T-test to 5 mM	0.5 mM vs 5 mM	ns	0.0031 **	< 0.001***	< 0.001 ***	< 0.001 ***
	5 mM + HS vs 5 mM	ns	ns	ns	ns	Ns
	0.5 mM + HS vs 5 mM	ns	0.032 *	< 0.001***	< 0.001 ***	< 0.001 ***
T-test to 0.5 mM	5 mM + HS vs 0.5 mM	ns	< 0.001 ***	< 0.001 ***	< 0.001 ***	< 0.001 ***
	0.5 mM + HS vs 0.5 mM	ns	ns	ns	0.011 *	0.0041**
T-test to 0.5 mM +HS	5 mM + HS vs 0.5 mM + HS	ns	0.011 *	< 0.001 ***	< 0.001 ***	< 0.001 ***

(b) Total root length

	Treatments	DAT 4	DAT 11	DAT 17	DAT 23	DAT 28
Total root length	5 mM	1.55 ± 0.15	3.98 ± 0.97	8.58 ± 1.74	13.46 ± 3.48	18.80 ± 4.11
(cm)	5 mM + HS	1.48 ± 0.14	4.85 ± 0.79	9.74 ± 1.83	14.82 ± 2.74	25.19 ± 4.85
	0.5 mM	1.58 ± 0.18	4.37 ± 0.91	8.96 ± 2.09	13.94 ± 3.90	22.12 ± 4.57
	0.5 mM + HS	1.53 ± 0.17	5.64 ± 1.39	11.72 ± 2.73	18.77 ± 3.32	27.75 ± 4.61
T test						
T-test to 5 mM	0.5 mM vs 5 mM	ns	ns	ns	ns	ns
	5 mM + HS vs 5 mM	ns	ns	ns	ns	0.011 *
	0.5 mM + HS vs 5 mM	ns	0.012 *	0.012 *	0.005 **	< 0.001 ***
T-test to 0.5 mM	5 mM + HS vs 0.5 mM	ns	ns	ns	ns	ns
	0.5 mM + HS vs 0.5 mM	ns	0.034 *	0.027 *	0.011 *	0.018 *
T-test to 0.5 mM +HS	5 mM + HS vs 0.5 mM + HS	ns	ns	ns	0.012 *	ns

(c) Primary root total length

	Treatments	DAT 4	DAT 11	DAT 17	DAT 23	DAT 28
Primary root total length	5 mM	1.55 ± 0.15	2.56 ± 0.41	4.08 ± 0.88	6.04 ± 0.79	7.41 ± 0.83
(cm)	5 mM + HS	1.48 ± 0.14	3.01 ± 0.41	5.16 ± 0.36	8.08 ± 0.85	9.64 ± 1.69
	0.5 mM	1.58 ± 0.18	3.16 ± 0.24	4.45 ± 0.64	6.70 ± 0.58	8.59 ± 1.15
	0.5 mM + HS	1.53 ± 0.17	3.76 ± 0.27	5.98 ± 0.74	9.40 ± 1.51	11.17 ± 1.23
T test						
T-test to 5 mM	0.5 mM vs 5 mM	ns	0.0017 **	ns	ns	0.035 *
	5 mM + HS vs 5 mM	ns	0.031 *	0.0032 **	< 0.001 ***	0.004 **
	0.5 mM + HS vs 5 mM	ns	< 0.001 ***	< 0.001 ***	< 0.001 ***	< 0.001 ***
T-test to 0.5 mM	5 mM + HS vs 0.5 mM	ns	ns	0.0088 **	< 0.001 ***	ns
	0.5 mM + HS vs 0.5 mM	ns	< 0.001 ***	< 0.001 ***	< 0.001 ***	< 0.001 ***
T-test to 0.5 mM +HS	5 mM + HS vs 0.5 mM + HS	ns	< 0.001 ***	0.0078 **	0.033 *	0.035 *

(d) Lateral root total length

	Treatments	DAT 4	DAT 11	DAT 17	DAT 23	DAT 28
Lateral root total length (cm)	5 mM	0	1.43 ± 0.95	4.50 ± 1.98	7.42 ± 3.55	11.39 ± 4.34
	5 mM +HS	0	1.21 ± 0.9	4.51 ± 2.38	7.25 ± 3.9	13.52 ± 4.23
	0.5 mM	0	1.79 ± 0.86	4.58 ± 1.53	7.27 ± 2.81	15.55 ± 4.82
	0.5 mM +HS	0	1.88 ± 1.23	5.75 ± 2.92	9.65 ± 4	16.58 ± 5.23
T test						
T-test to 5 mM	0.5 mM vs 5 mM	ns	ns	ns	ns	ns
	5 mM +HS vs 5 mM	ns	ns	ns	ns	ns
	0.5 mM +HS vs 5 mM	ns	ns	ns	ns	0.05 *
T-test to 0.5 mM	5 mM +HS vs 0.5 mM	ns	ns	ns	ns	ns
	0.5 mM +HS vs 0.5 mM	ns	ns	ns	ns	ns
T-test to 0.5 mM +HS	5 mM +HS vs 0.5 mM + HS	ns	ns	ns	ns	ns

(e) Root hair length

	Treatments	DAT 4	DAT 11	DAT 17	DAT 23	DAT 28
Root hair length (cm)	5 mM	0.52 ± 0.09	0.49 ± 0.07	0.39 ± 0.06	0.31 ± 0.04	0.26 ± 0.05
	5 mM +HS	0.45 ± 0.11	0.50 ± 0.09	0.33 ± 0.04	0.26 ± 0.04	0.18 ± 0.06
	0.5 mM	0.68 ± 0.04	0.74 ± 0.09	0.60 ± 0.05	0.55 ± 0.02	0.54 ± 0.06
	0.5 mM +HS	0.74 ± 0.08	0.69 ± 0.09	0.56 ± 0.09	0.42 ± 0.07	0.40 ± 0.09
T test						
T-test to 5 mM	0.5 mM vs 5 mM	< 0.001 ***	< 0.001 ***	< 0.001 ***	< 0.001 ***	< 0.001 ***
	5 mM +HS vs 5 mM	ns	ns	0.027 *	0.015 *	0.0086 **
	0.5 mM +HS vs 5 mM	< 0.001 ***	< 0.001 ***	< 0.001 ***	0.0023 **	< 0.001 ***
T-test to 0.5 mM	5 mM +HS vs 0.5 mM	< 0.001 ***	< 0.001 ***	< 0.001 ***	< 0.001 ***	< 0.001 ***
	0.5 mM +HS vs 0.5 mM	ns	ns	ns	< 0.001 ***	< 0.001 ***
T-test to 0.5 mM +HS	5 mM +HS vs 0.5 mM + HS	< 0.001 ***	< 0.001 ***	< 0.001 ***	< 0.001 ***	< 0.001 ***

Table S3. Ammonium and nitrate depletion in the EcoFAB medium, showed in Fig. 4a-b (n=5). Asterisk indicate statistically differences according to unpaired t-test at the 0.05 level. ns, no significant; *, p < 0.05; **, p < 0.01; ***, p < 0.001; ***. (a) ammonium depletion; (b) nitrate depletion.

(a) Ammonium depletion

	Treatments	DAT 4	DAT 8	DAT 11	DAT 14	DAT 17
Ammonium depletion	5 mM	4.70 ± 1.9	5.89 ± 1.01	7.48 ± 0.67	7.59 ± 1.34	10.73 ± 0.78
(µmol)	5 mM + HS	5.26 ± 1.61	3.09 ± 0.83	4.32 ± 1.61	6.61 ± 1.28	9.76 ± 2.20
	0.5 mM	1.24 ± 0.29	1.65 ± 0.25	1.43 ± 0.31	1.28 ± 0.11	1.56 ± 0.15
	0.5 mM + HS	1.52 ± 0.17	1.67 ± 0.15	1.59 ± 0.15	1.45 ± 0.21	1.65 ± 0.3
t-test						
t-test to 5 mM	0.5 mM vs 5 mM	0.007**	< 0.001***	< 0.001***	< 0.001***	< 0.001***
	5 mM + HS vs 5 mM	ns	0.0021**	0.0042**	ns	ns
	0.5 mM + HS vs 5 mM	0.01*	< 0.001***	< 0.001***	< 0.001***	< 0.001***
t-test to 0.5 mM	5 mM + HS vs 0.5 mM	< 0.001***	0.0062**	0.0044380	< 0.001***	< 0.001***
	0.5 mM + HS vs 0.5 mM	ns	ns	ns	ns	ns
t-test to 0.5 mM +HS	5 mM + HS vs 0.5 mM + HS	< 0.001***	0.0063**	0.0044**	< 0.001***	< 0.001***

	Treatments	DAT 20	DAT 23	DAT 26	DAT 28
Ammonium depletion	5 mM	10.62 ± 1.77	10.74 ± 2.4	9.52 ± 0.66	6.97 ± 1.99
(µmol)	5 mM + HS	11.34 ± 1.83	9.70 ± 1.64	8.74 ± 1.73	6.70 ± 2.27
	0.5 mM	1.64 ± 0.11	1.73 ± 0.12	1.65 ± 0.13	1.71 ± 0.22
	0.5 mM + HS	1.89 ± 0.15	1.87 ± 0.13	1.89 ± 0.07	1.90 ± 0.06
t-test					
t-test to 5 mM	0.5 mM vs 5 mM	< 0.001***	< 0.001***	< 0.001***	< 0.001***
	5 mM + HS vs 5 mM	ns	ns	ns	ns
	0.5 mM + HS vs 5 mM	< 0.001***	< 0.001***	< 0.001***	0.001**
t-test to 0.5 mM	5 mM + HS vs 0.5 mM	< 0.001***	< 0.001***	< 0.001***	0.0012**
	0.5 mM + HS vs 0.5 mM	0.031*	ns	0.0122	ns
t-test to 0.5 mM +HS	5 mM + HS vs 0.5 mM + HS	< 0.001***	< 0.001***	< 0.001***	0.0015**

(b) Nitrate depletion

	Treatments	DAT 4	DAT 8	DAT 11	DAT 14	DAT 17
Nitrate depletion	5 mM	1.20 ± 1.01	2.55 ± 1.53	3.91 ± 1.17	3.10 ± 2.13	8.49 ± 1.19
(µmol)	5 mM + HS	2.19 ± 2.36	0.70 ± 1	0.12 ± 0.26	1.68 ± 1.3	8.10 ± 2.92
	0.5 mM	0.92 ± 0.27	1.44 ± 0.38	1.43 ± 0.43	1.34 ± 0.22	1.57 ± 0.16
	0.5 mM + HS	0.93 ± 0.35	1.53 ± 0.39	1.73 ± 0.15	1.56 ± 0.16	1.64 ± 0.34
t-test						
t-test to 5 mM	0.5 mM vs 5 mM	ns	ns	0.004**	ns	< 0.001***
	5 mM + HS vs 5 mM	ns	ns	< 0.001***	ns	ns
	0.5 mM + HS vs 5 mM	ns	ns	0.0061**	ns	< 0.001***
t-test to 0.5 mM	5 mM + HS vs 0.5 mM	ns	ns	< 0.001***	ns	0.0011**
	0.5 mM + HS vs 0.5 mM	ns	ns	ns	ns	ns
t-test to 0.5 mM +HS	5 mM + HS vs 0.5 mM + HS	ns	ns	< 0.001***	ns	0.0012**

	Treatments	DAT 20	DAT 23	DAT 26	DAT 28
Nitrate depletion	5 mM	6.57 ± 2.78	8.64 ± 3.28	5.50 ± 0.67	3.60 ± 2.3
(µmol)	5 mM + HS	7.75 ± 1.23	6.24 ± 3.35	3.41 ± 2.8	2.33 ± 2.45
	0.5 mM	1.66 ± 0.19	1.79 ± 0.13	1.76 ± 0.13	1.85 ± 0.15
	0.5 mM + HS	1.88 ± 0.16	1.90 ± 0.14	1.96 ± 0.09	1.94 ± 0.08
t-test					
t-test to 5 mM	0.5 mM vs 5 mM	0.0078**	0.0031**	< 0.001***	ns
	5 mM + HS vs 5 mM	ns	ns	ns	ns
	0.5 mM + HS vs 5 mM	0.0097**	0.0034**	< 0.001***	ns
t-test to 0.5 mM	5 mM + HS vs 0.5 mM	0.0011**	< 0.001***	0.018*	ns
	0.5 mM + HS vs 0.5 mM	ns	ns	ns	0.034*
t-test to 0.5 mM +HS	5 mM + HS vs 0.5 mM + HS	0.0012**	< 0.001***	0.02*	ns

Table S4 Cumulative N depletion in the EcoFAB medium, showed in Fig. 5 (n=5). Asterisk indicate statistically differences according to unpaired t-test at the 0.05 level. ns, no significant; *, p < 0.05; **, p < 0.01; **, p < 0.001; ***. (a) cumulative N depletion; (b) cumulative ammonium depletion; (c) cumulative nitrate depletion; (d) Delta N , (in cases of different variance unpaired t-test with Welch's correction was taken).

(a) Cumulative N depletion

	Treatments	DAT 4	DAT 8	DAT 11	DAT 14	DAT 17
Cumulative N depletion	5 mM	13.37 ± 2.55	21.81 ± 3.92	33.20 ± 3.3	43.88 ± 4.96	63.10 ± 6.59
(µmol)	5 mM + HS	14.91 ± 3.92	18.70 ± 4.05	23.14 ± 3.27	31.43 ± 3.69	49.28 ± 4.37
	0.5 mM	9.62 ± 0.55	12.71 ± 0.66	15.57 ± 1.28	18.19 ± 1.48	21.32 ± 1.62
	0.5 mM + HS	9.91 ± 0.41	13.12 ± 0.84	16.44 ± 0.98	19.44 ± 1.14	22.73 ± 1.71
t-test						
t-test to 5 mM	0.5 mM vs 5 mM	0.021*	0.0018**	< 0.001***	< 0.001***	< 0.001***
	5 mM + HS vs 5 mM	ns	ns	0.0018**	0.0031**	0.0069**
	0.5 mM + HS vs 5 mM	0.028*	0.0025**	< 0.001***	< 0.001***	< 0.001***
t-test to 0.5 mM	5 mM + HS vs 0.5 mM	0.018*	0.012*	0.0015**	< 0.001***	0.018*
	0.5 mM + HS vs 0.5 mM	ns	ns	ns	ns	ns
t-test to 0.5 mM +HS	5 mM + HS vs 0.5 mM + HS	0.022*	0.017*	0.0025**	< 0.001***	0.022*

	Treatments	DAT 20	DAT 23	DAT 26	DAT 28
Cumulative N depletion	5 mM	80.29 ± 8.53	99.68 ± 11.43	114.70 ± 11.9	125.27 ±15.01
(µmol)	5 mM + HS	68.38 ± 6.13	84.33 ± 3.93	96.48 ± 5.13	105.51 ± 8.62
	0.5 mM	24.63 ± 1.72	28.14 ± 1.87	31.55 ± 1.93	35.11 ± 1.86
	0.5 mM + HS	26.49 ± 1.94	30.27 ± 2.01	34.12 ± 2.03	37.96 ± 2.14
t-test					
t-test to 5 mM	0.5 mM vs 5 mM	< 0.001***	< 0.001***	< 0.001***	< 0.001***
	5 mM + HS vs 5 mM	0.047*	0.033*	0.021*	0.047*
	0.5 mM + HS vs 5 mM	< 0.001***	< 0.001***	< 0.001***	< 0.001***
t-test to 0.5 mM	5 mM + HS vs 0.5 mM	< 0.001***	< 0.001***	< 0.001***	< 0.001***
	0.5 mM + HS vs 0.5 mM	ns	ns	ns	ns
t-test to 0.5 mM +HS	5 mM + HS vs 0.5 mM + HS	< 0.001***	< 0.001***	< 0.001***	< 0.001***

(b) Cumulative ammonium depletion

	Treatments	DAT 4	DAT 8	DAT 11	DAT 14	DAT 17
Cumulative ammonium depletion (µmol)	5 mM	4.70 ± 1.9	10.59 ± 2.33	18.07 ± 2.03	25.66 ± 2.49	36.39 ± 3.12
	5 mM + HS	5.26 ± 1.61	8.34 ± 1.06	12.67 ± 1.18	19.28 ± 1.85	29.03 ± 1.05
	0.5 mM	1.24 ± 0.29	2.89 ± 0.29	4.32 ± 0.56	5.60 ± 0.59	7.16 ± 0.64
	0.5 mM + HS	1.52 ± 0.17	3.19 ± 0.23	4.78 ± 0.36	6.23 ± 0.56	7.88 ± 0.72
t-test						
t-test to 5 mM	0.5 mM vs 5 mM	0.007**	< 0.001***	< 0.001***	< 0.001***	< 0.001***
	5 mM + HS vs 5 mM	ns	ns	0.0015**	0.0027**	0.0019**
	0.5 mM + HS vs 5 mM	0.01*	< 0.001***	< 0.001***	< 0.001***	< 0.001***
t-test to 0.5 mM	5 mM + HS vs 0.5 mM	< 0.001***	< 0.001***	< 0.001***	< 0.001***	< 0.001***
	0.5 mM + HS vs 0.5 mM	ns	ns	ns	ns	ns
t-test to 0.5 mM +HS	5 mM + HS vs 0.5 mM + HS	< 0.001***	< 0.001***	< 0.001***	< 0.001***	< 0.001***

	Treatments	DAT 20	DAT 23	DAT 26	DAT 28
Cumulative ammonium depletion (µmol)	5 mM	47.01 ± 4.11	57.76 ± 5.58	67.28 ± 5.57	74.24 ± 7.12
	5 mM + HS	40.38 ± 1.82	50.08 ± 2.1	58.82 ± 2.4	65.52 ± 4.43
	0.5 mM	8.80 ± 0.68	10.53 ± 0.75	12.18 ± 0.74	13.89 ± 0.74
	0.5 mM + HS	9.77 ± 0.8	11.63 ± 0.83	13.53 ± 0.85	15.43 ± 0.9
t-test					
t-test to 5 mM	0.5 mM vs 5 mM	< 0.001***	< 0.001***	< 0.001***	< 0.001***
	5 mM + HS vs 5 mM	0.017*	0.031*	0.022*	ns
	0.5 mM + HS vs 5 mM	< 0.001***	< 0.001***	< 0.001***	< 0.001***
t-test to 0.5 mM	5 mM + HS vs 0.5 mM	< 0.001***	< 0.001***	< 0.001***	< 0.001***
	0.5 mM + HS vs 0.5 mM	ns	ns	0.045*	0.031*
t-test to 0.5 mM +HS	5 mM + HS vs 0.5 mM + HS	< 0.001***	< 0.001***	< 0.001***	< 0.001***

(c) Cumulative nitrate depletion

	Treatments	DAT 4	DAT 8	DAT 11	DAT 14	DAT 17
Cumulative nitrate depletion (μmol)	5 mM	1.20 ± 1.01	3.75 ± 1.85	7.66 ± 1.71	10.76 ± 3.07	19.25 ± 3.89
	5 mM + HS	2.19 ± 2.36	2.89 ± 3.28	3.01 ± 3.18	4.68 ± 3.23	12.78 ± 4.13
	0.5 mM	0.92 ± 0.27	2.36 ± 0.41	3.78 ± 0.77	5.12 ± 0.92	6.70 ± 1.02
	0.5 mM + HS	0.93 ± 0.35	2.46 ± 0.67	4.19 ± 0.71	5.75 ± 0.76	7.38 ± 1.1
t-test						
t-test to 5 mM	0.5 mM vs 5 mM	ns	ns	0.0032**	0.0079**	< 0.001***
	5 mM + HS vs 5 mM	ns	ns	0.023*	0.02*	0.043*
	0.5 mM + HS vs 5 mM	ns	ns	0.0056**	0.013*	< 0.001***
t-test to 0.5 mM	5 mM + HS vs 0.5 mM	ns	ns	ns	ns	0.013*
	0.5 mM + HS vs 0.5 mM	ns	ns	ns	ns	ns
t-test to 0.5 mM +HS	5 mM + HS vs 0.5 mM + HS	ns	ns	ns	ns	0.023*

	Treatments	DAT 20	DAT 23	DAT 26	DAT 28
Cumulative nitrate depletion (μmol)	5 mM	25.82 ± 4.79	34.46 ± 6.10	39.96 ± 6.54	43.56 ± 8.06
	5 mM + HS	20.54 ± 5.07	26.78 ± 2.70	30.19 ± 3.86	32.52 ± 4.97
	0.5 mM	8.35 ± 1.07	10.14 ± 1.15	11.90 ± 1.21	13.75 ± 1.15
	0.5 mM + HS	9.26 ± 1.22	11.17 ± 1.26	13.12 ± 1.26	15.06 ± 1.31
t-test					
t-test to 5 mM	0.5 mM vs 5 mM	< 0.001***	< 0.001***	< 0.001***	< 0.001***
	5 mM + HS vs 5 mM	ns	ns	0.03*	0.043*
	0.5 mM + HS vs 5 mM	< 0.001***	< 0.001***	< 0.001***	< 0.001***
t-test to 0.5 mM	5 mM + HS vs 0.5 mM	< 0.001***	< 0.001***	< 0.001***	< 0.001***
	0.5 mM + HS vs 0.5 mM	ns	ns	ns	ns
t-test to 0.5 mM +HS	5 mM + HS vs 0.5 mM + HS	0.0014**	< 0.001***	< 0.001***	< 0.001***

(d) Delta N, W indicates t-test using Welch's correction

	Treatments	Sample 1	Sample 2	Sample 3	Sample 4	Sample 5	Mean ± SD
Delt N	5 mM	8.98	-3.21	10.38	29.20	-7.75	7.521 ± 14.39
(μmol)	5 mM + HS	-6.93	-5.59	-30.47	-14.74	-9.71	-13.49 ± 10.12
	0.5 mM	16.34	8.89	11.49	9.39	16.64	12.55 ± 3.731
	0.5 mM + HS	16.81	12.67	16.13	18.36	9.60	14.71 ± 3.536
t-test							
t-test to 5 mM	0.5 mM vs 5 mM (W)						ns
	5 mM + HS vs 5 mM						0.028*
	0.5 mM + HS vs 5 mM (W)						ns
t-test to 0.5 mM	5 mM + HS vs 0.5 mM (W)						0.0028**
	0.5 mM + HS vs 0.5 mM						ns
t-test to 0.5 mM +HS	5 mM + HS vs 0.5 mM + HS (W)						0.0021**

Reference:

Frank, J. A., Reich, C. I., Sharma, S., Weisbaum, J. S., Wilson, B. A., & Olsen, G. J. (2008). Critical evaluation of two primers commonly used for amplification of bacterial 16S rRNA genes. Applied and environmental microbiology, 74(8), 2461-2470.

Zhou, X., Wu, X., Li, T., Jia, M., Liu, X., Zou, Y., ... & Wen, F. (2018). Identification, characterization, and expression analysis of auxin response factor (ARF) gene family in Brachypodium distachyon. *Functional & integrative genomics*, *18*(6), 709-724.

Wang, J., Hüner, N., & Tian, L. (2019). Identification and molecular characterization of the Brachypodium distachyon NRT2 family, with a major role of BdNRT2. 1. *Physiologia plantarum*, *165*(3), 498-510.
